# Supplementary figures and images for: Repulsive Sema3E-Plexin-D1 signaling coordinates both axonal extension and steering via activating an autoregulatory factor, Mtss1
Source: eLife. 2024 Mar 25;13:e96891. doi: 10.7554/eLife.96891 (PMC11001299; doi:10.7554/eLife.96891)

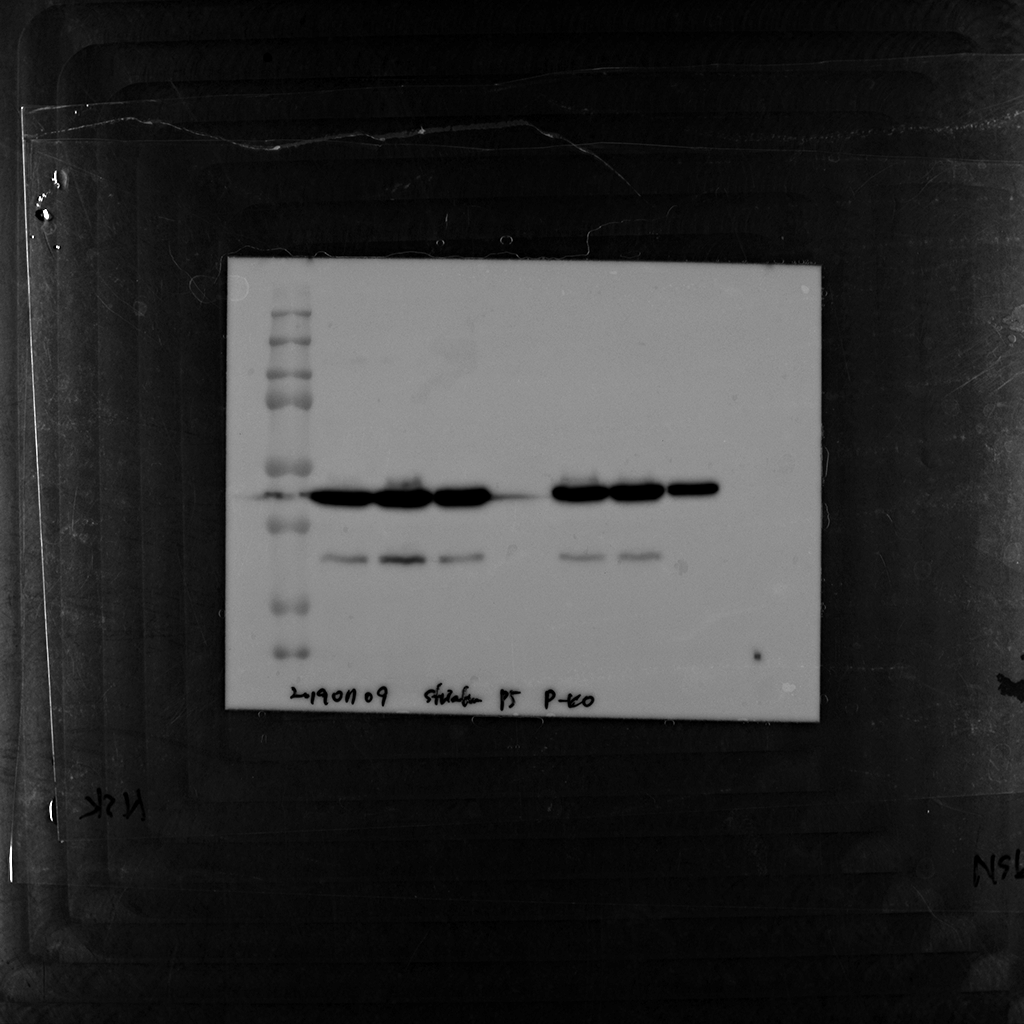

Supplement: Figure 1—source data 1. [file elife-96891-fig1-data1.zip › Figure 1_source data 1/Fig1D-actin.tif]

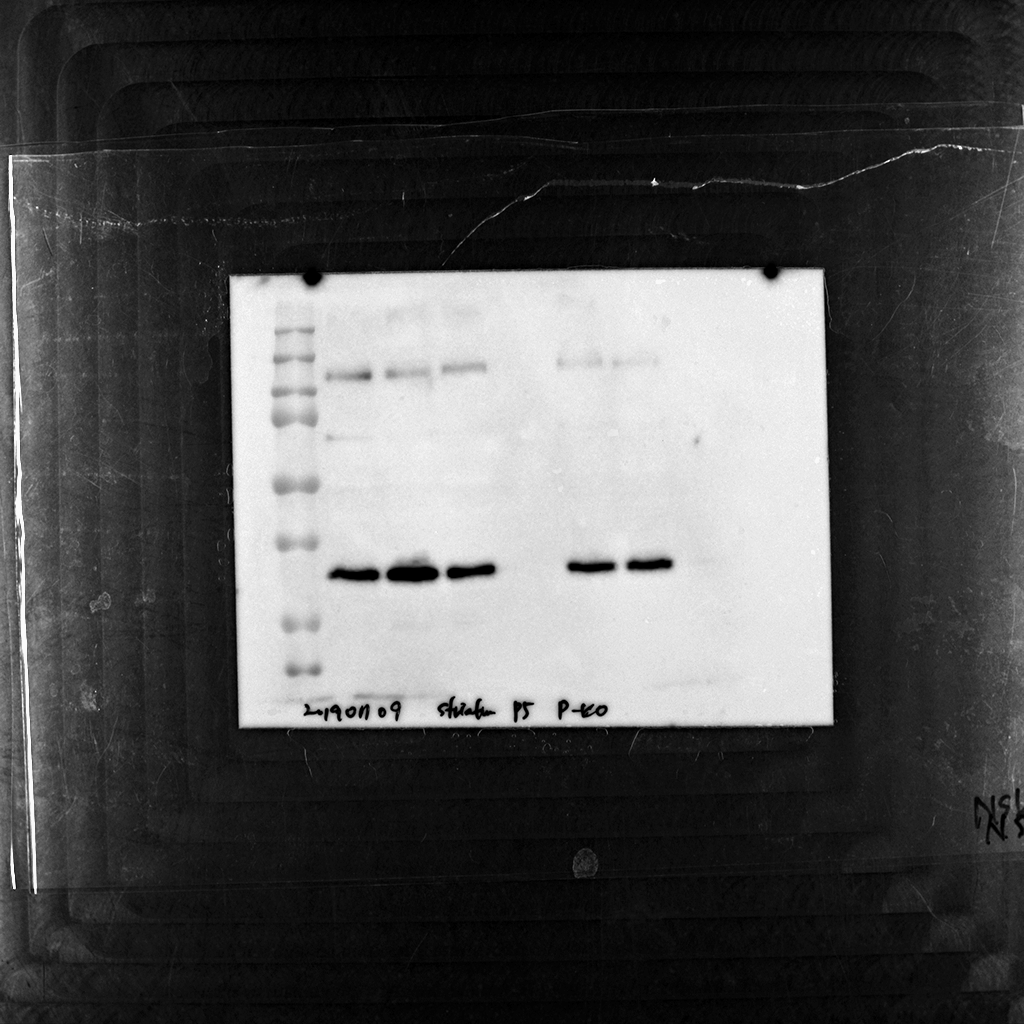

Supplement: Figure 1—source data 1. [file elife-96891-fig1-data1.zip › Figure 1_source data 1/Fig1D-Mtss1.tif]

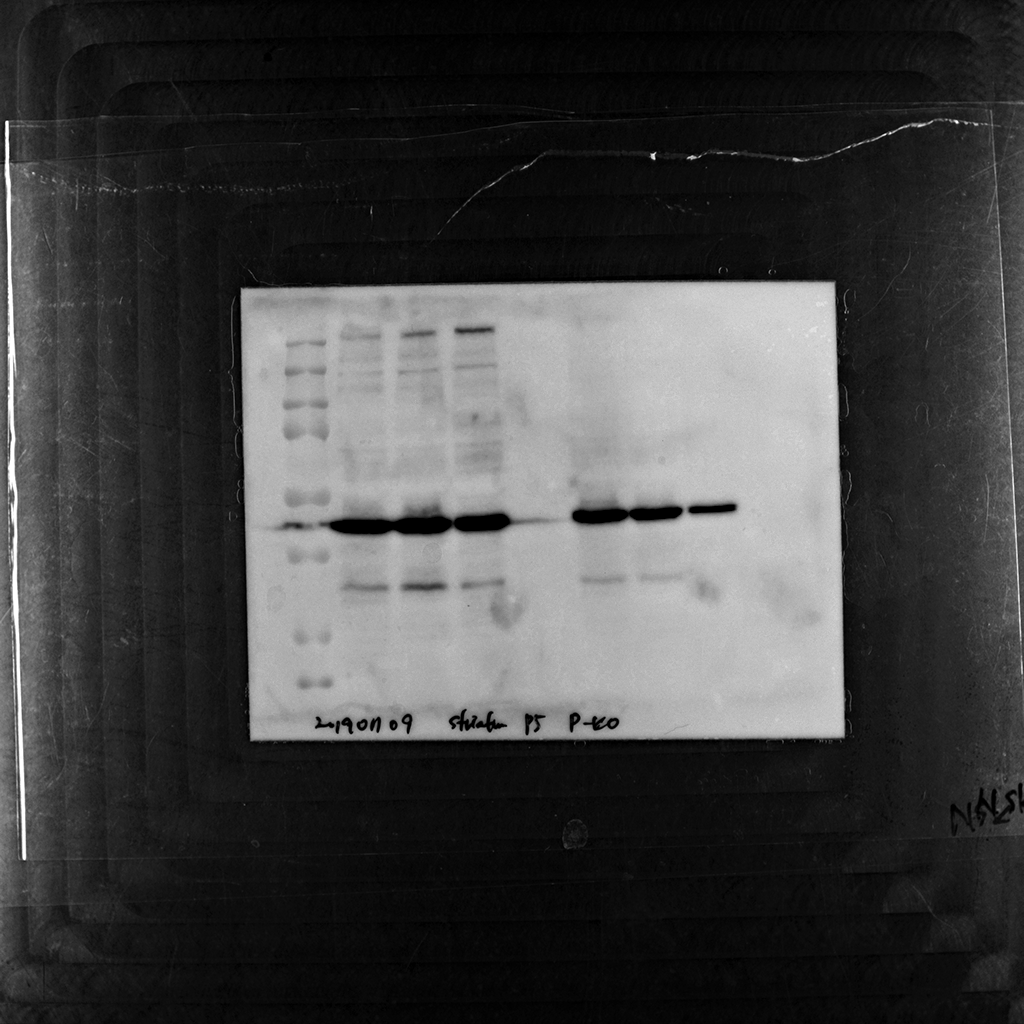

Supplement: Figure 1—source data 1. [file elife-96891-fig1-data1.zip › Figure 1_source data 1/Fig1D-Plexin-D1.tif]

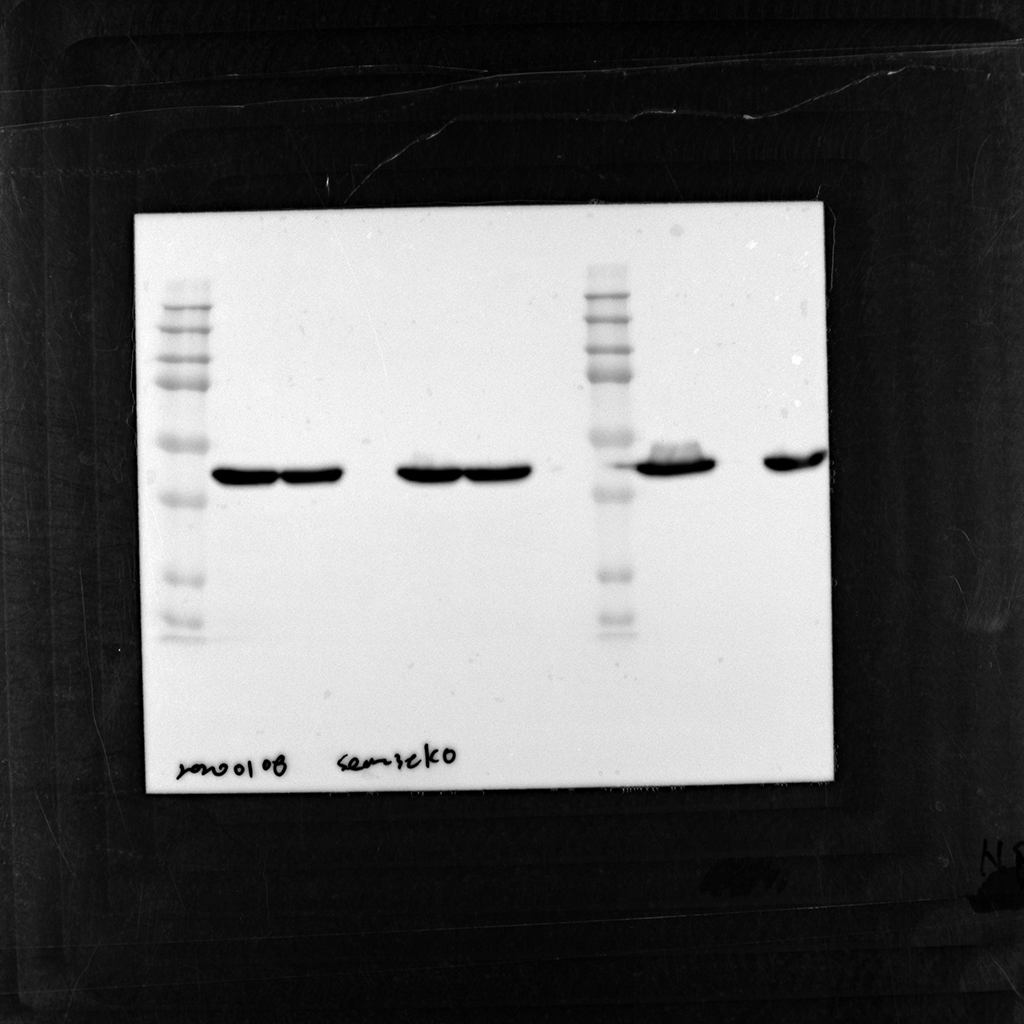

Supplement: Figure 1—source data 1. [file elife-96891-fig1-data1.zip › Figure 1_source data 1/Fig1F-actin.tif]

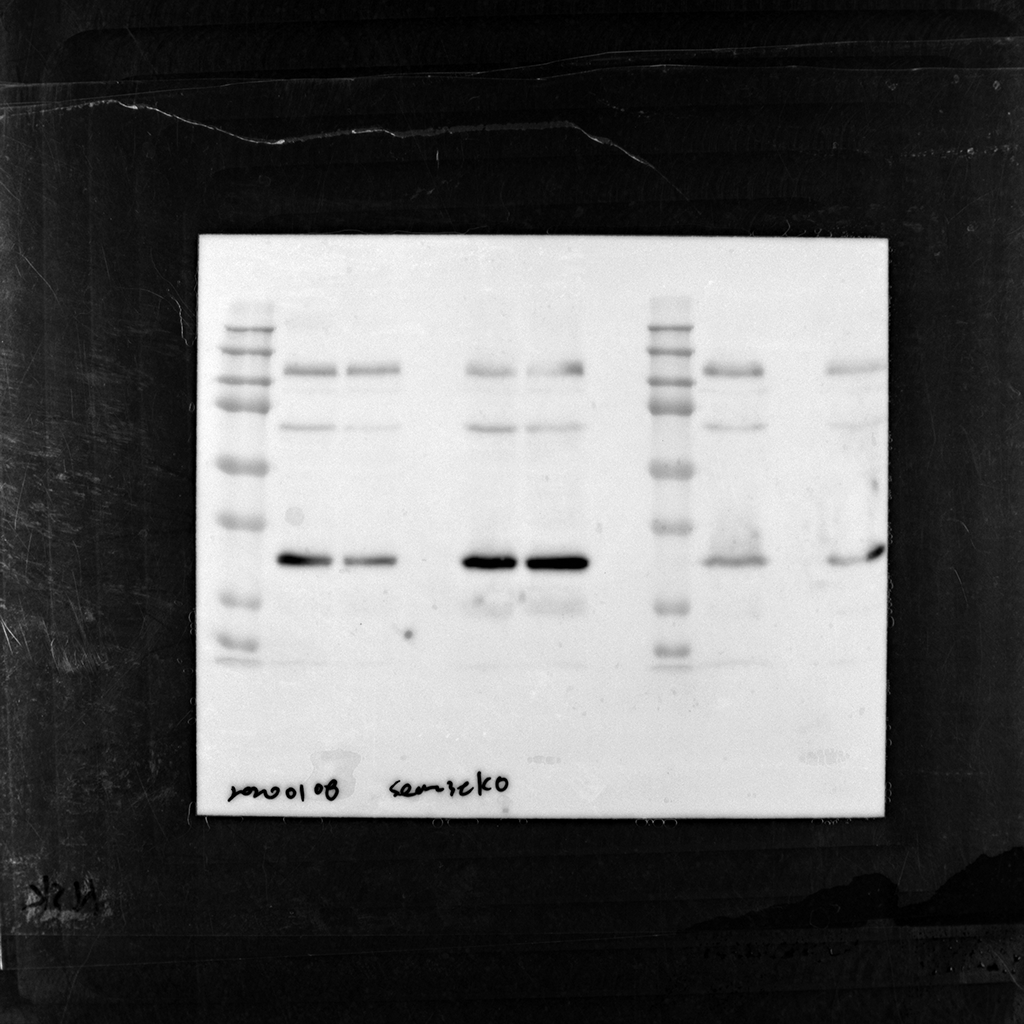

Supplement: Figure 1—source data 1. [file elife-96891-fig1-data1.zip › Figure 1_source data 1/Fig1F-Mtss1.tif]

Figure 1 – source data 1 (panel D)

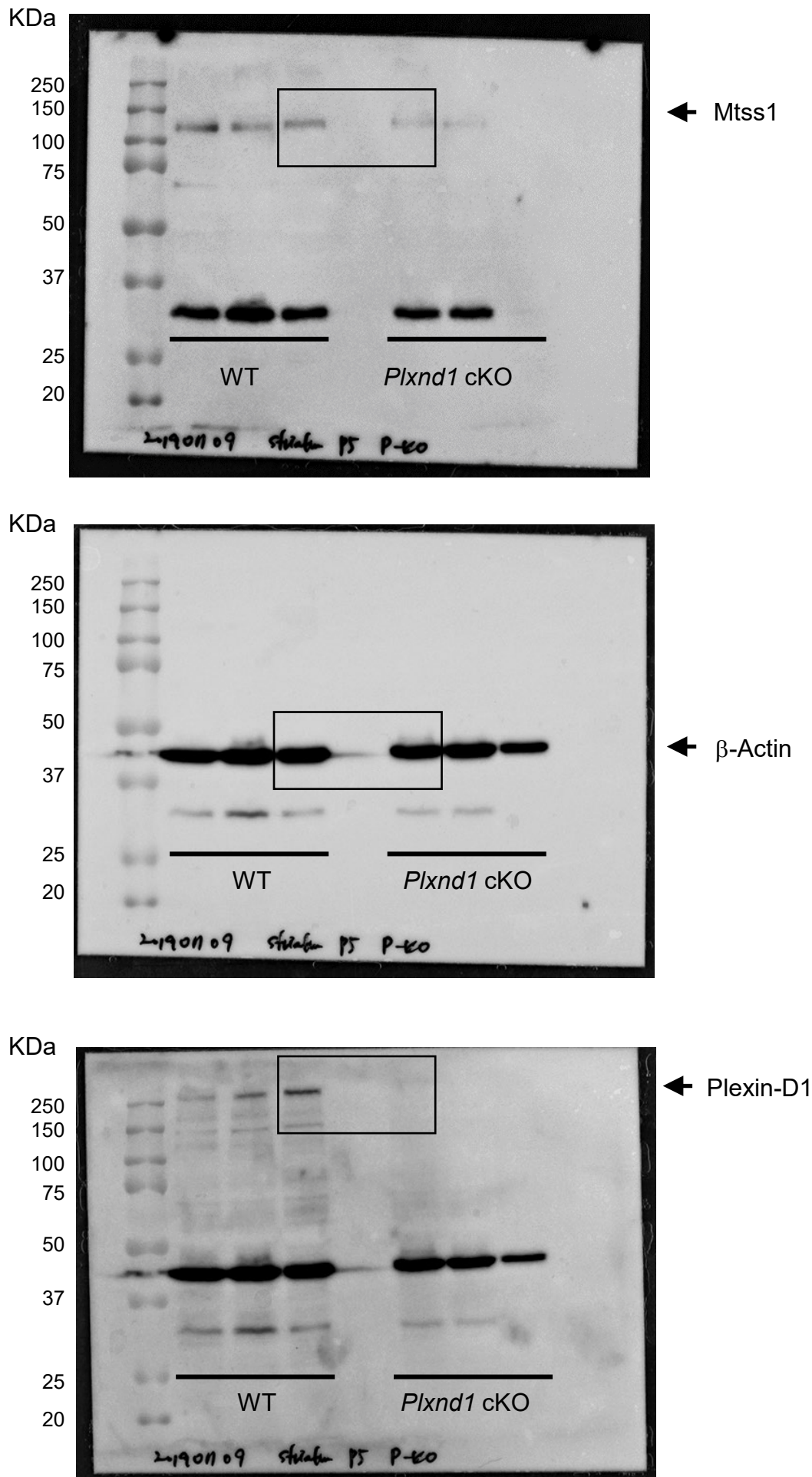

Supplement: Figure 1—source data 1. [file elife-96891-fig1-data1.zip › Figure 1_source data 1/Figure 1_labelled_1D.pdf]

Figure 1 – source data 1 (panel F)

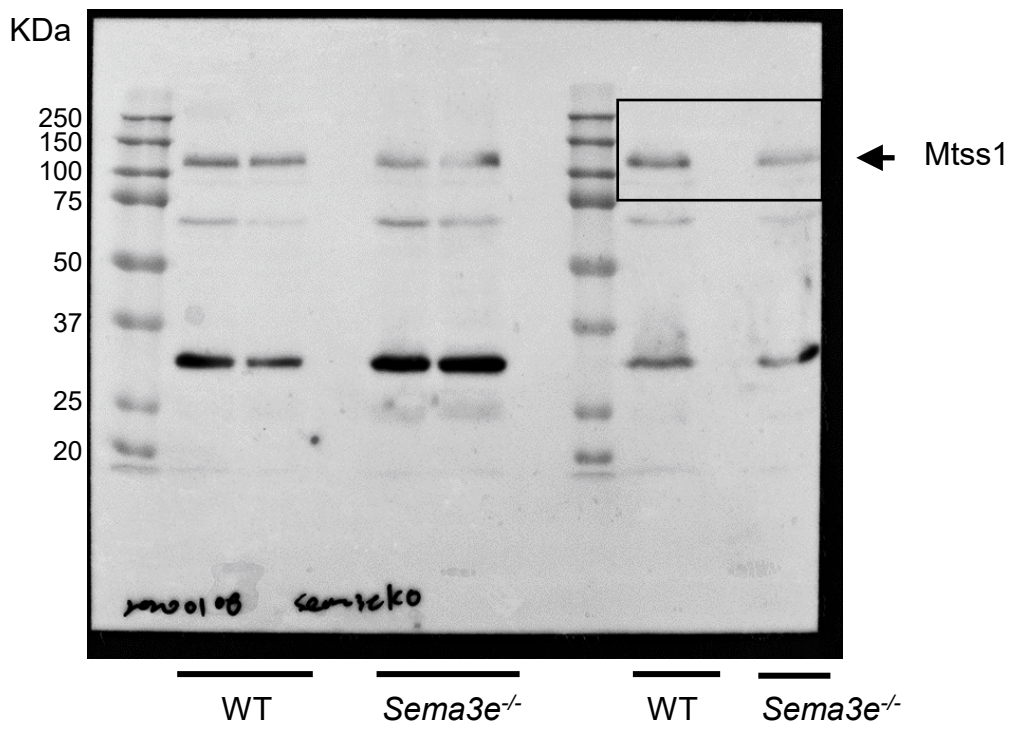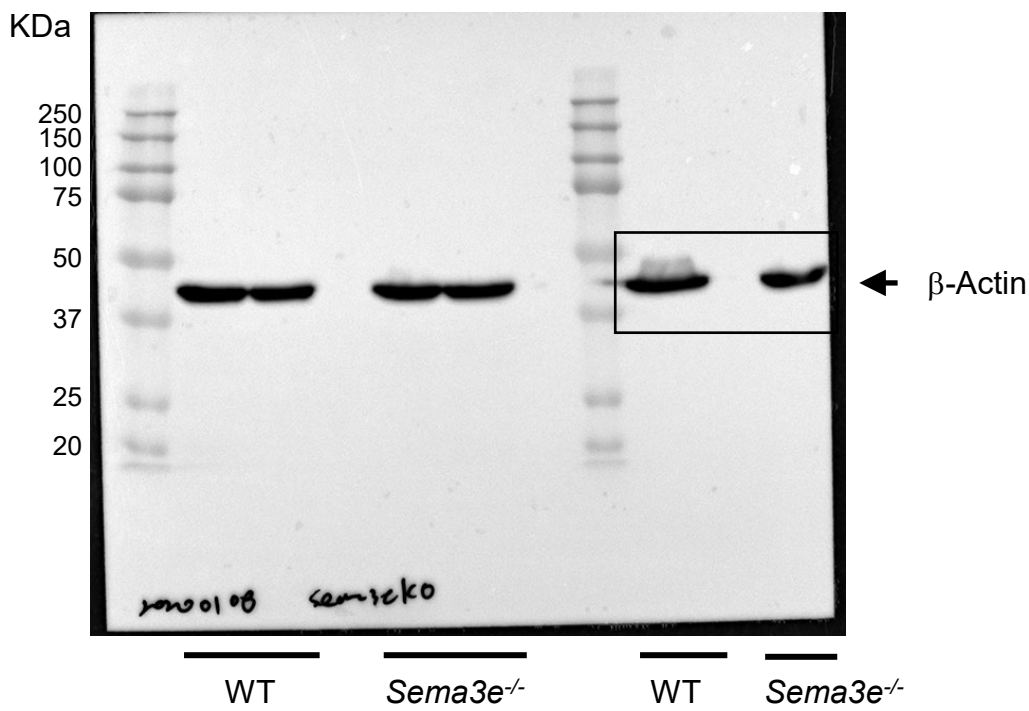

Supplement: Figure 1—source data 1. [file elife-96891-fig1-data1.zip › Figure 1_source data 1/Figure 1_labelled_1F.pdf]

Figure 1 – source data 1 (panel K)

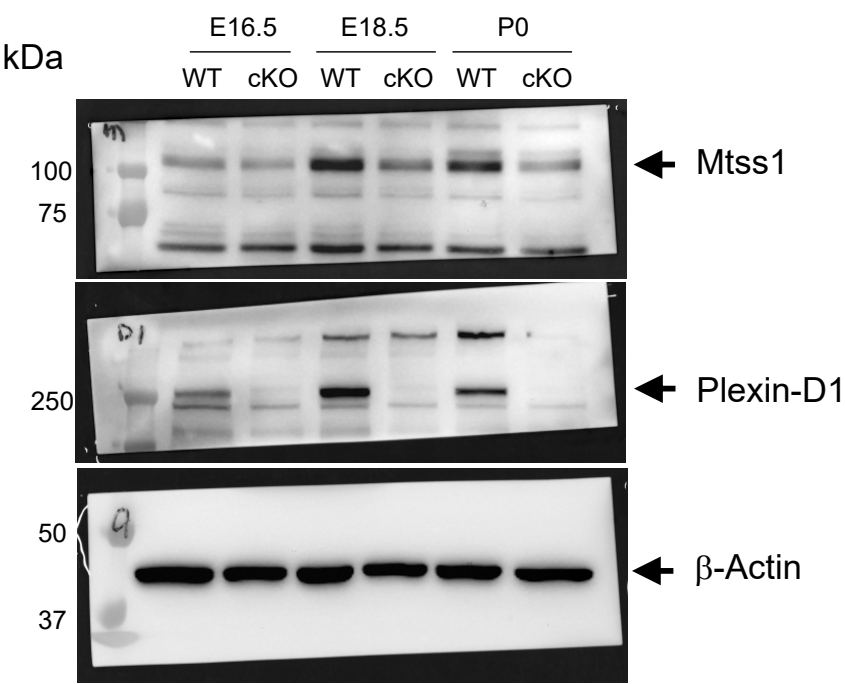

Supplement: Figure 1—source data 1. [file elife-96891-fig1-data1.zip › Figure 1_source data 1/Figure 1_labelled_1K.pdf]

Figure 1 – source data 1 (panel M)

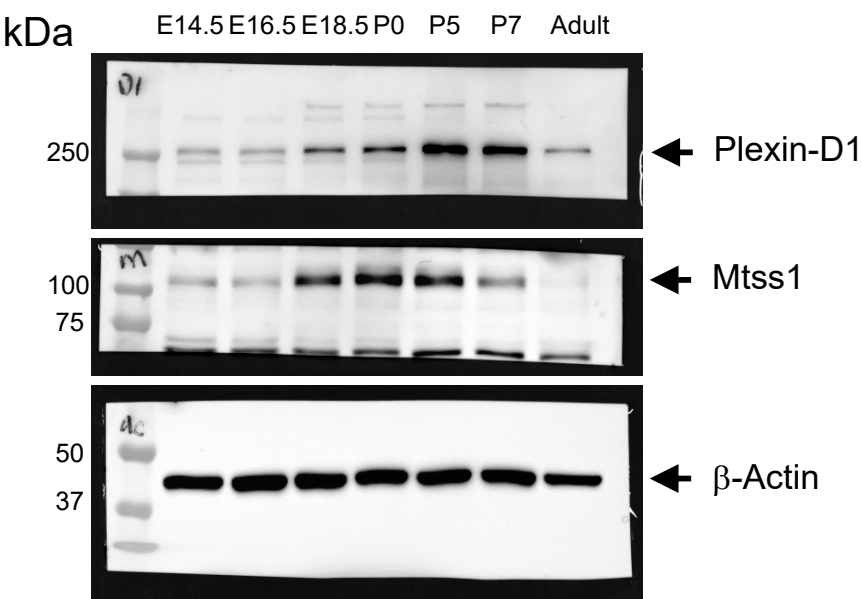

Supplement: Figure 1—source data 1. [file elife-96891-fig1-data1.zip › Figure 1_source data 1/Figure 1_labelled_1M.pdf]

Supplementary Figure 1 – source data 1 (panel B)

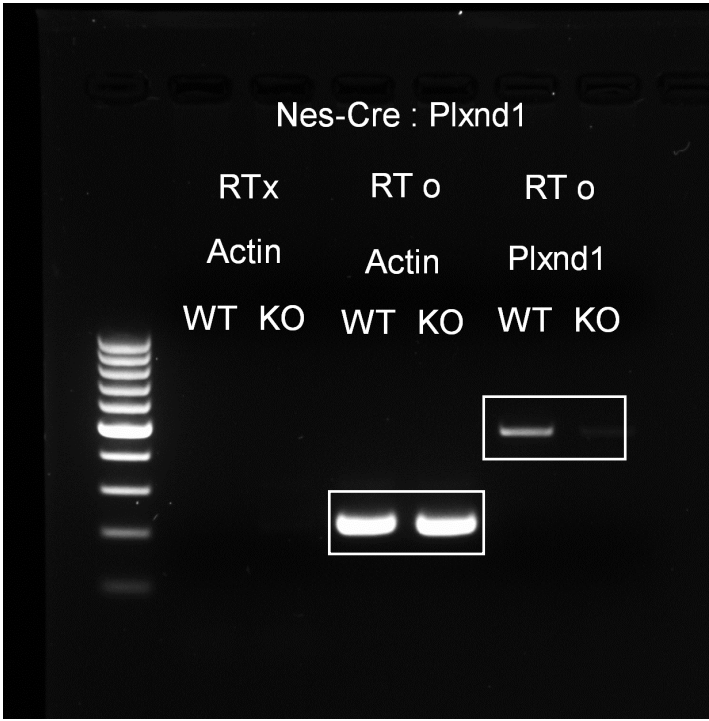

Supplement: Figure 1—figure supplement 1—source data 1. [file elife-96891-fig1-figsupp1-data1.zip › Figure 1_supplementary Figure 1_source data 1/Figure S1_labelled_S1B.pdf]

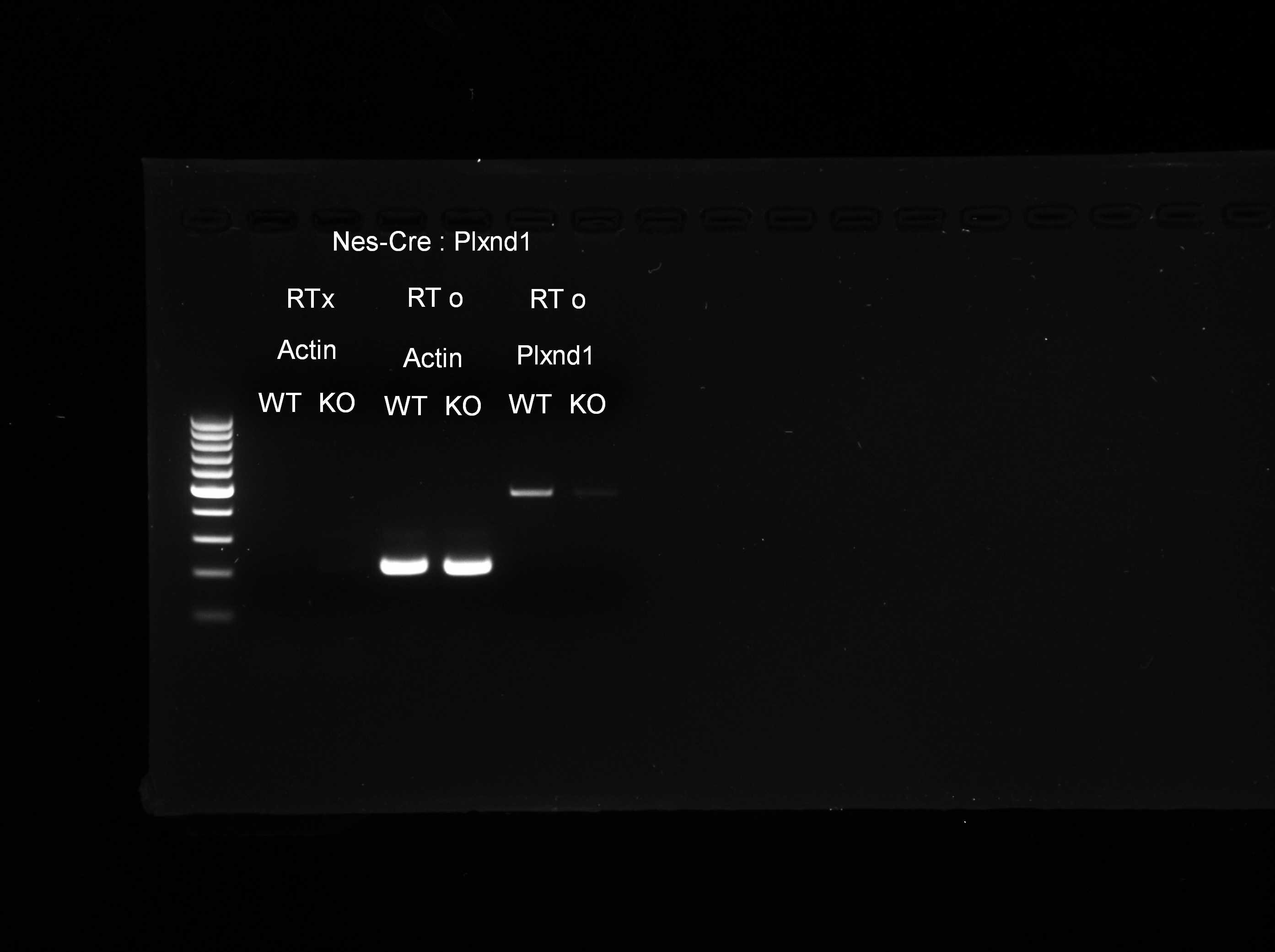

Supplement: Figure 1—figure supplement 1—source data 1. [file elife-96891-fig1-figsupp1-data1.zip › Figure 1_supplementary Figure 1_source data 1/Supple Fig2C_confirm NesCre Plxnd1 (WT,KO).tif]

Supplementary Figure 2 – source data 1 (panel C)

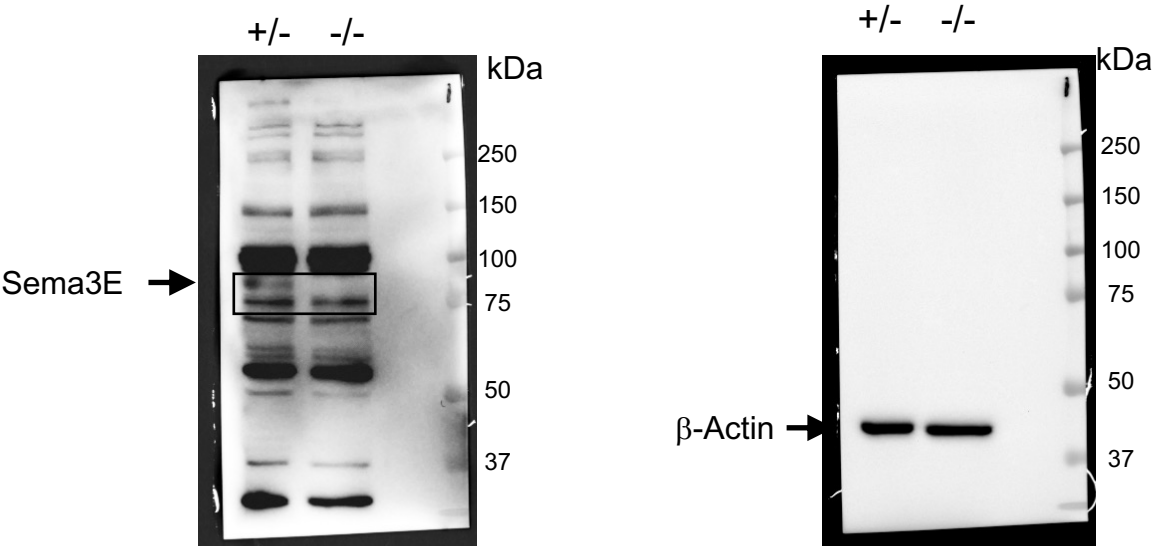

Supplement: Figure 1—figure supplement 2—source data 1. [file elife-96891-fig1-figsupp2-data1.zip › Figure 1_supplementary Figure 2_source data 1/Figure S2_labelled_S2C.pdf]

Figure 2 – source data 1 (panel A)

2A

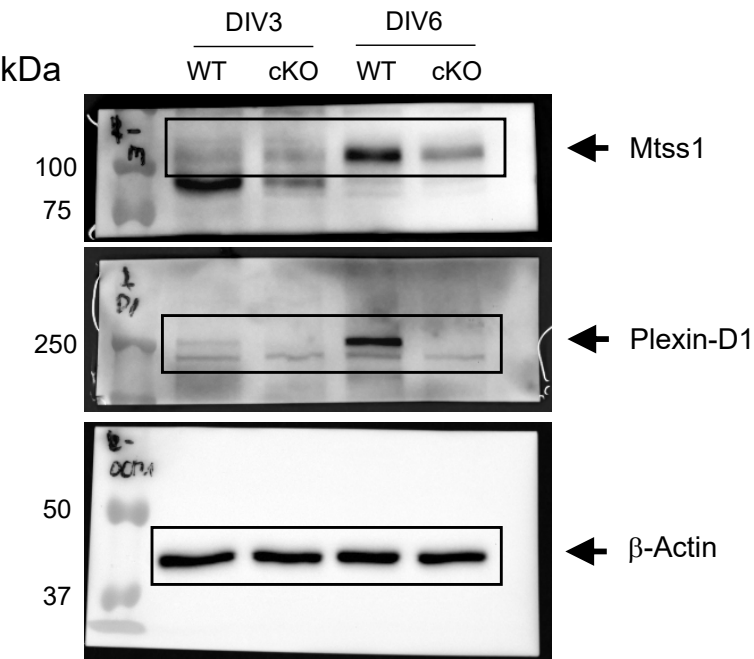

Supplement: Figure 2—source data 1. [file elife-96891-fig2-data1.zip › Figure 2_source data 1/Figure 2_labelled_2A.pdf]

Figure 2 – source data 1 (panel C)

2C

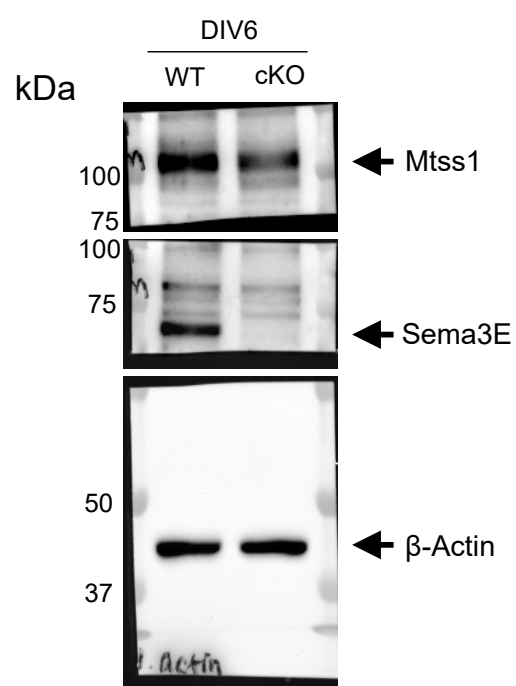

Supplement: Figure 2—source data 1. [file elife-96891-fig2-data1.zip › Figure 2_source data 1/Figure 2_labelled_2C.pdf]

Figure 2 – source data 1 (panel F)

2F

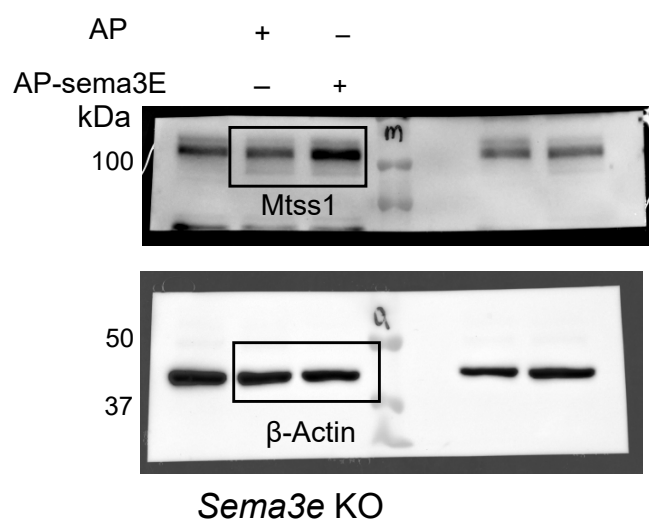

Supplement: Figure 2—source data 1. [file elife-96891-fig2-data1.zip › Figure 2_source data 1/Figure 2_labelled_2F.pdf]

Figure 2 – source data 1 (panel G)

2G

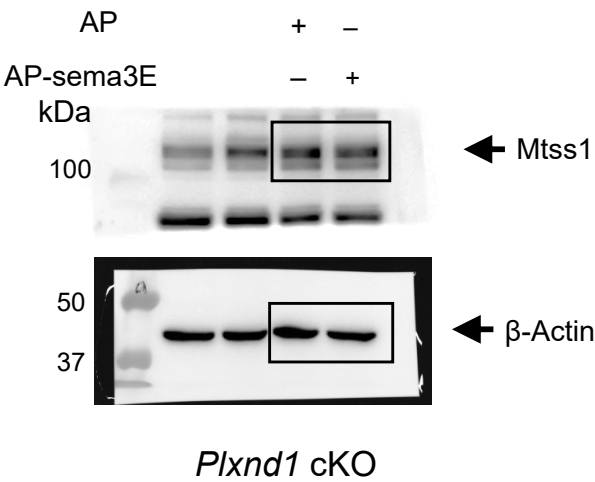

Supplement: Figure 2—source data 1. [file elife-96891-fig2-data1.zip › Figure 2_source data 1/Figure 2_labelled_2G.pdf]

Figure 2 – source data 1 (panel J)

2J

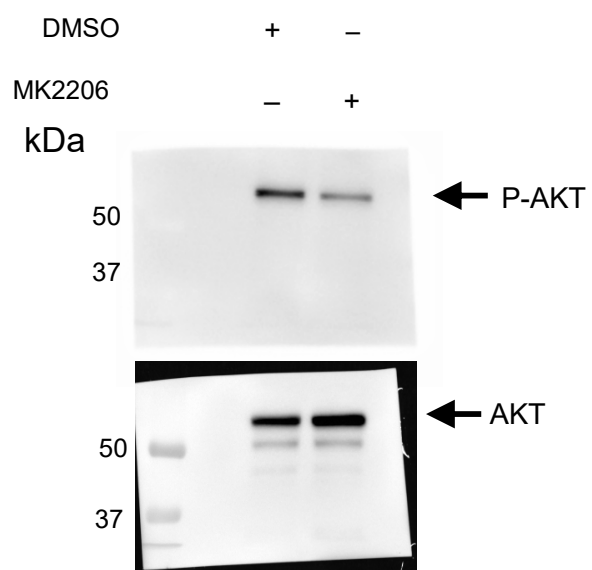

Supplement: Figure 2—source data 1. [file elife-96891-fig2-data1.zip › Figure 2_source data 1/Figure 2_labelled_2J.pdf]

Figure 2 – source data 1 (panel K)

2K

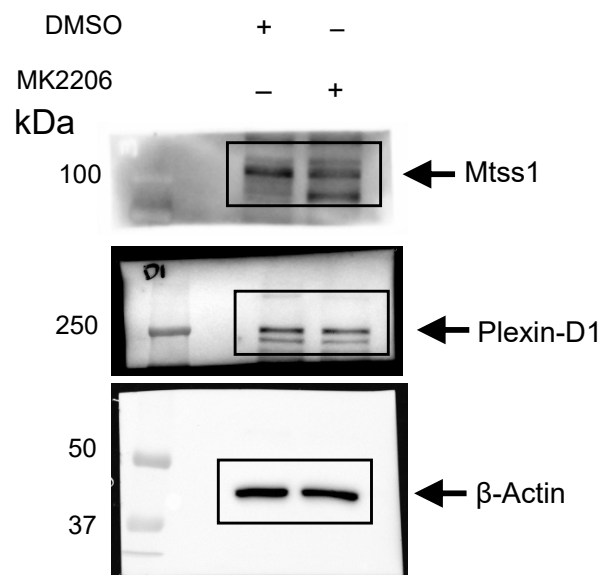

Supplement: Figure 2—source data 1. [file elife-96891-fig2-data1.zip › Figure 2_source data 1/Figure 2_labelled_2K.pdf]

Figure 2 – source data 1 (panel N)

2N

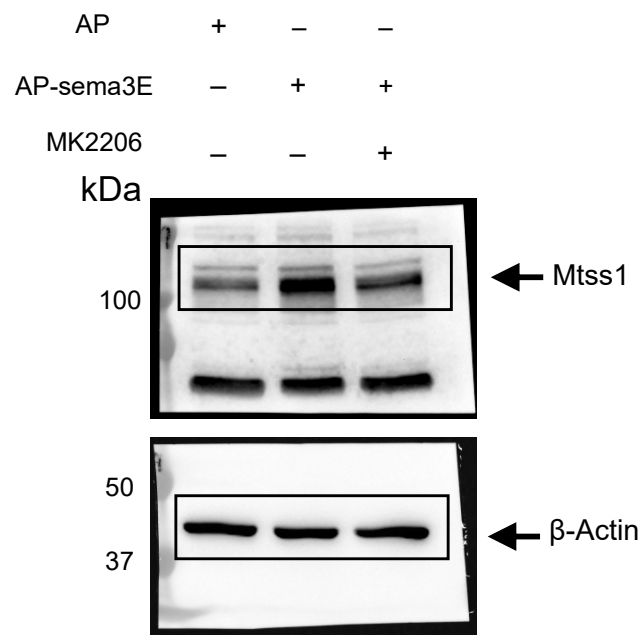

Supplement: Figure 2—source data 1. [file elife-96891-fig2-data1.zip › Figure 2_source data 1/Figure 2_labelled_2N.pdf]

Supplementary Figure 3 – source data 1 (panel A)

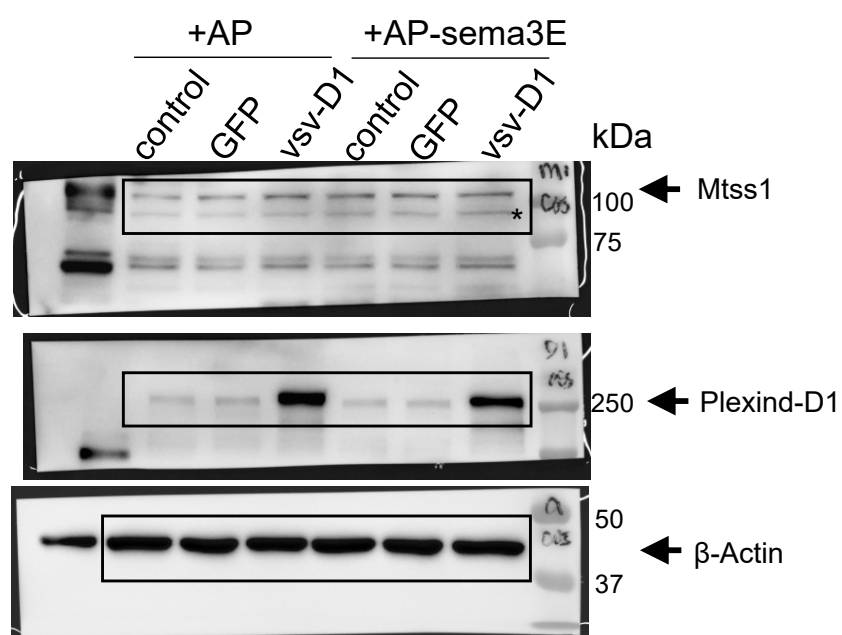

Supplement: Figure 3—figure supplement 1—source data 1. [file elife-96891-fig3-figsupp1-data1.zip › Figure 3_supplementary Figure 3_source data 1/Figure S3_labelled_S3A.pdf]

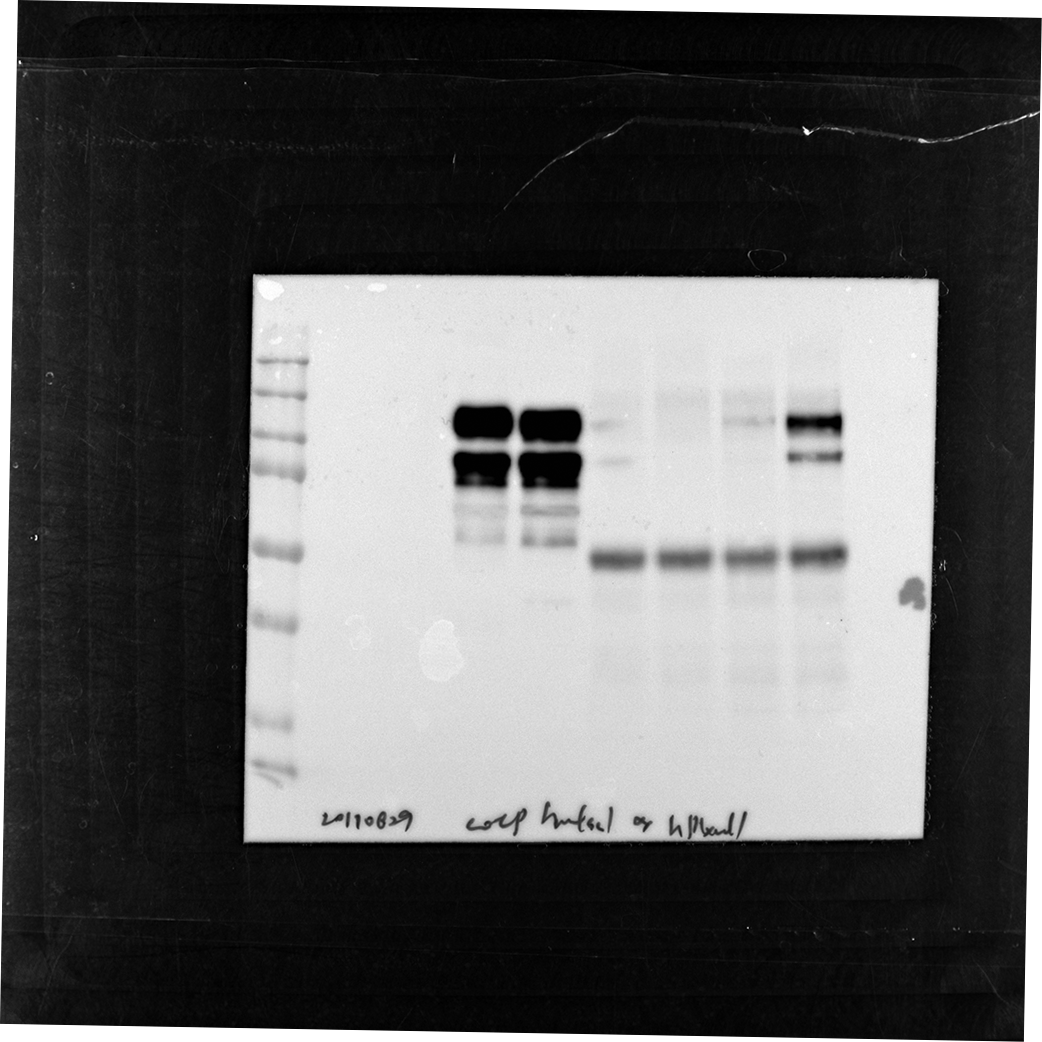

Supplement: Figure 4—source data 1. [file elife-96891-fig4-data1.zip › Figure 4_source data 1/Fig 4B_vsv IP_Mtss1-myc.tif]

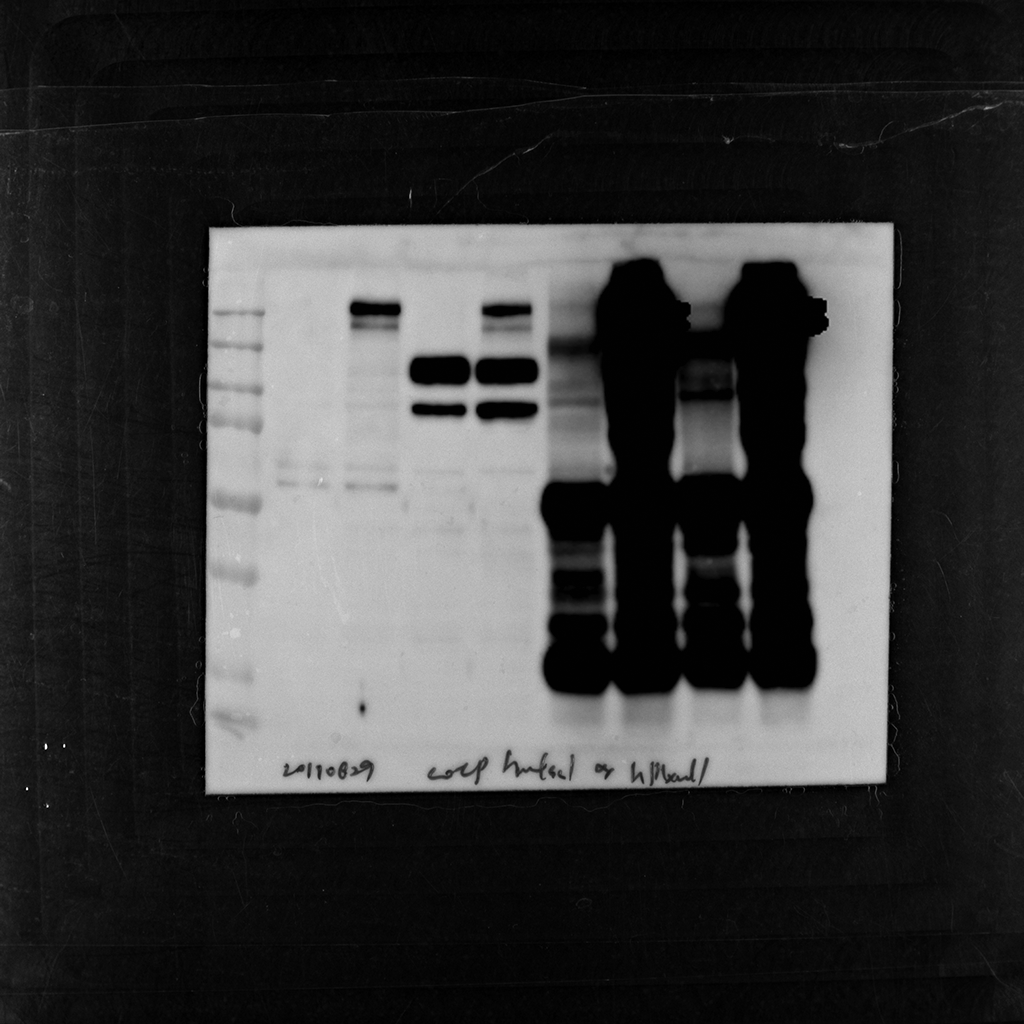

Supplement: Figure 4—source data 1. [file elife-96891-fig4-data1.zip › Figure 4_source data 1/Fig 4B_vsv IP_vsv-Plexin-D1_input.tif]

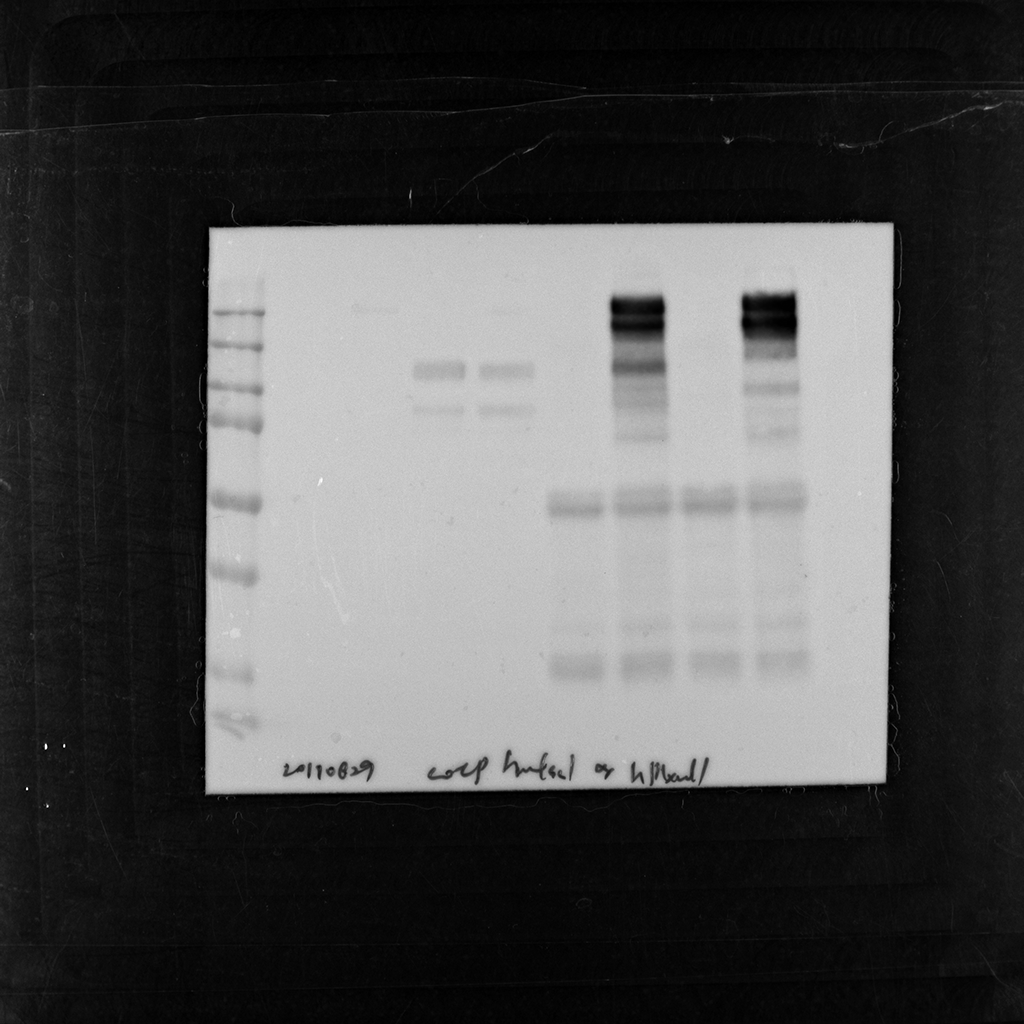

Supplement: Figure 4—source data 1. [file elife-96891-fig4-data1.zip › Figure 4_source data 1/Fig 4B_vsv IP_vsv-Plexin-D1_IP.tif]

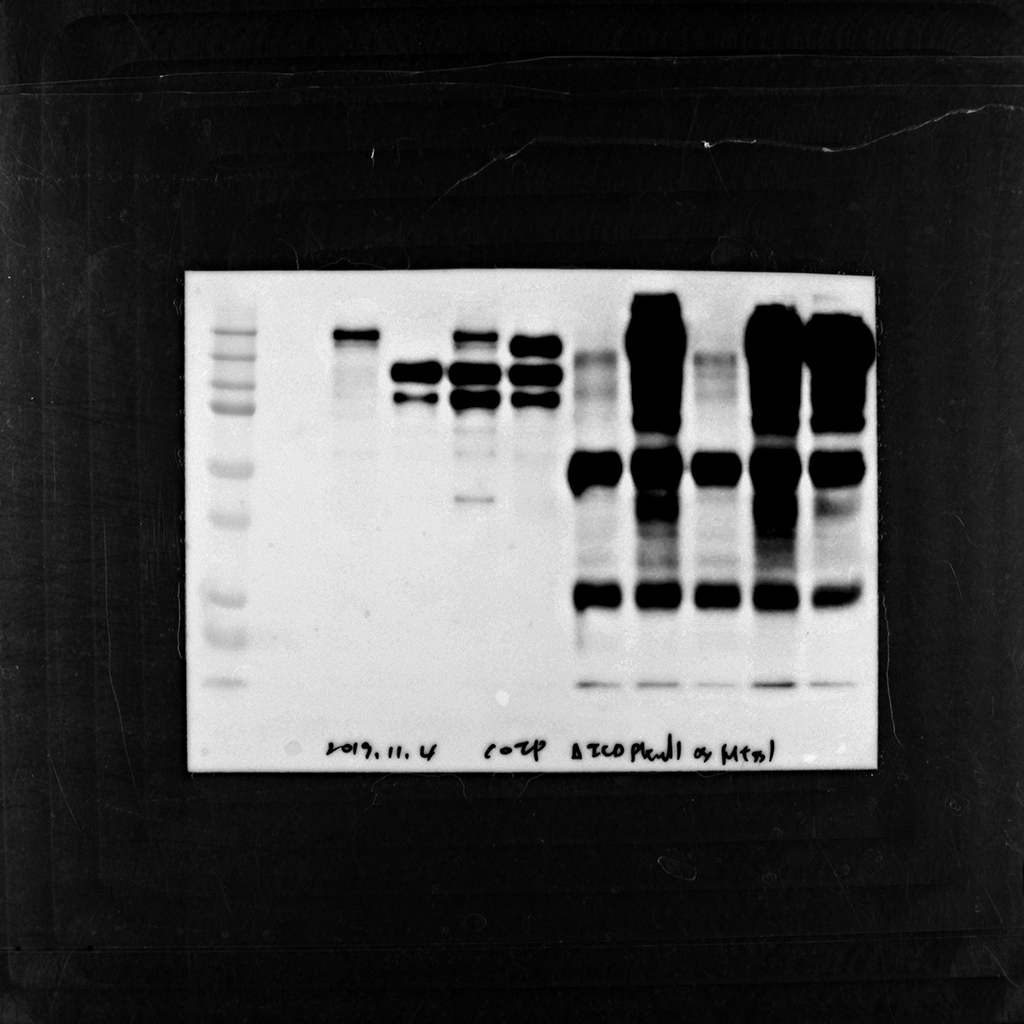

Supplement: Figure 4—source data 1. [file elife-96891-fig4-data1.zip › Figure 4_source data 1/Fig 4C-vsv-Plexin-D1_input.tif]

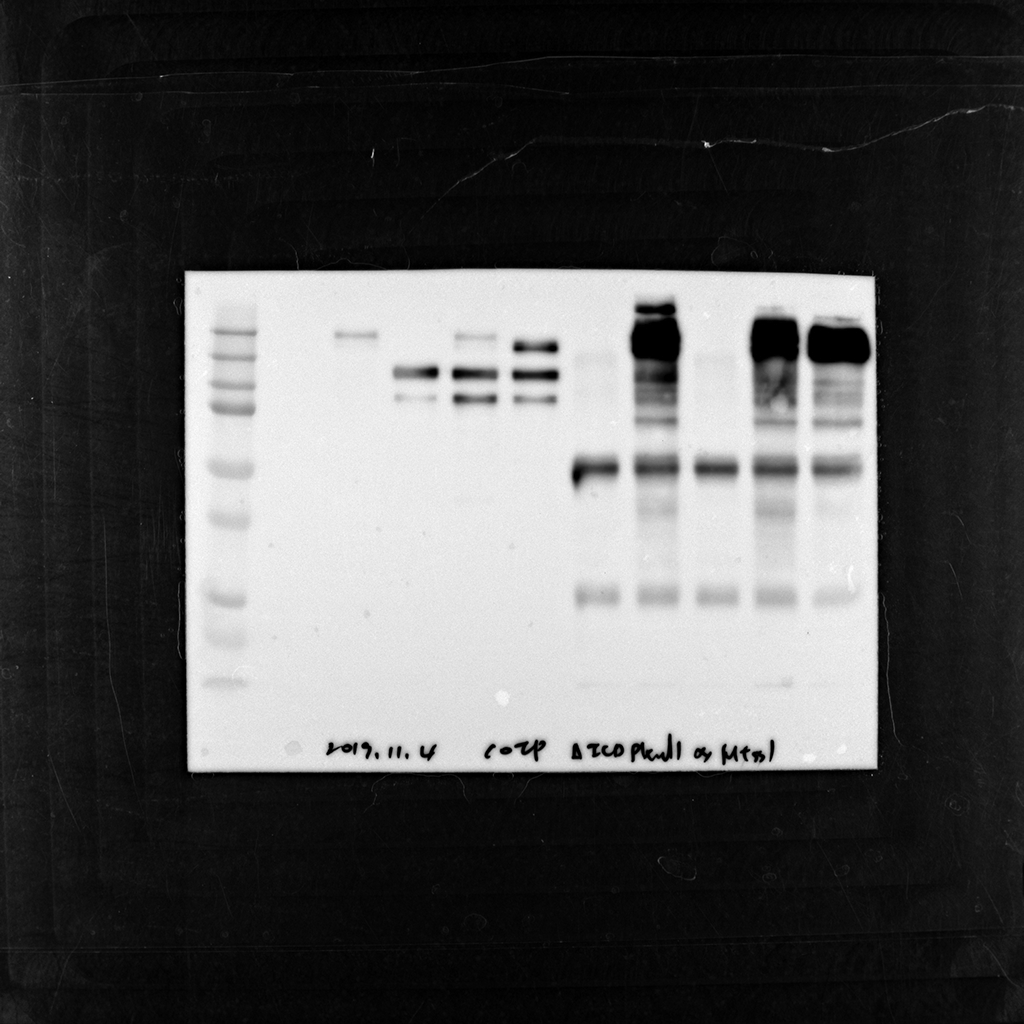

Supplement: Figure 4—source data 1. [file elife-96891-fig4-data1.zip › Figure 4_source data 1/Fig 4C-vsv-Plexin-D1_IP.tif]

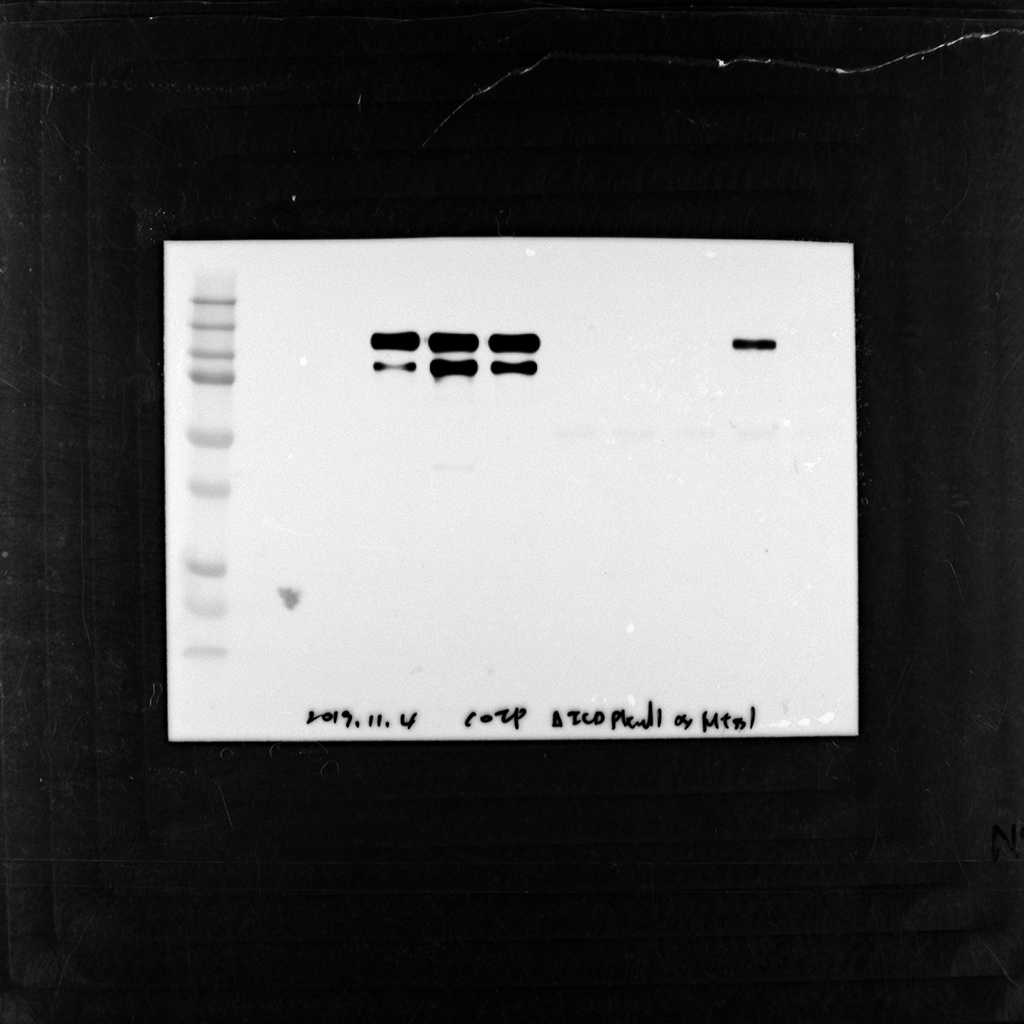

Supplement: Figure 4—source data 1. [file elife-96891-fig4-data1.zip › Figure 4_source data 1/Fig 4C_Mtss1-myc.tif]

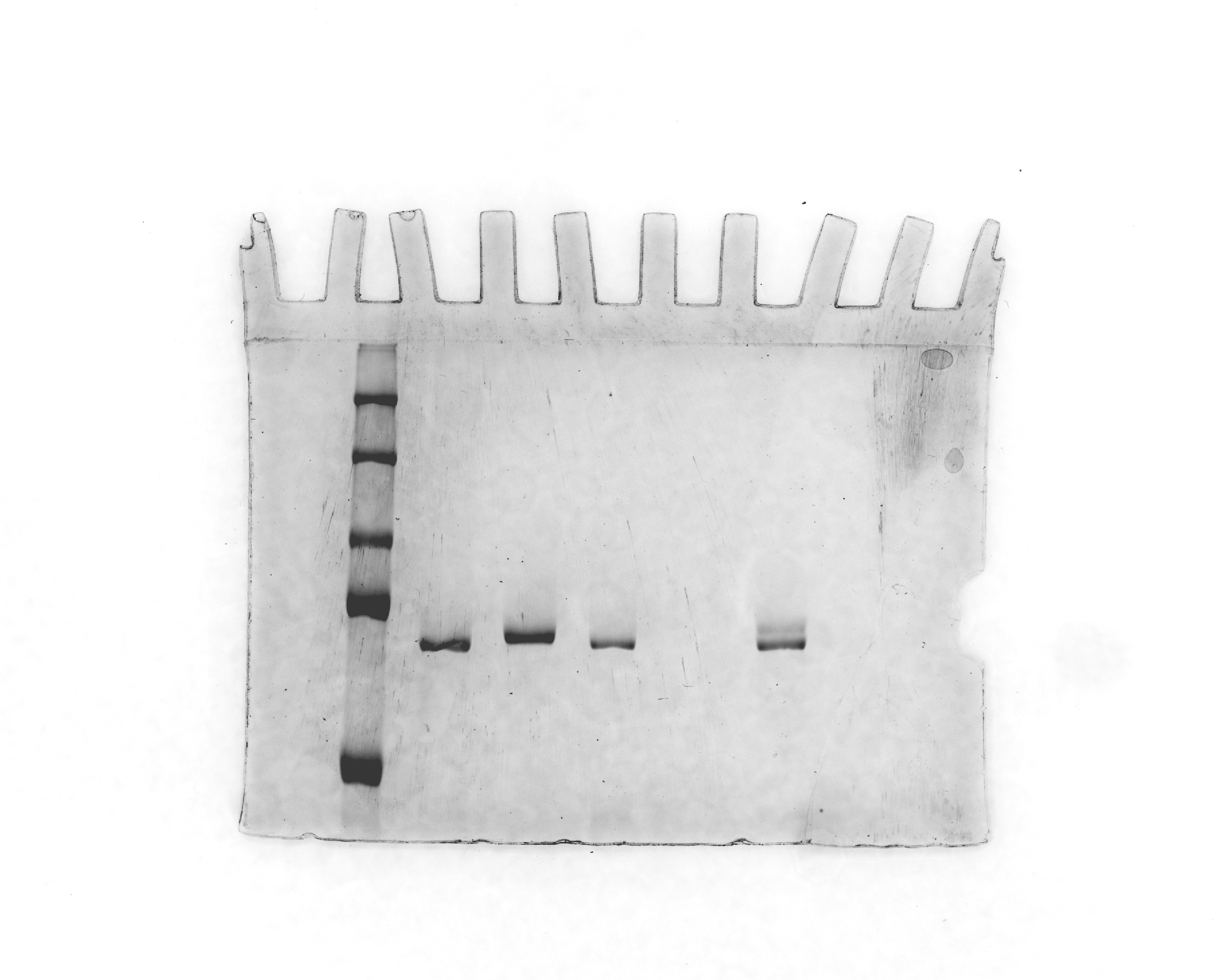

Supplement: Figure 4—source data 1. [file elife-96891-fig4-data1.zip › Figure 4_source data 1/Fig 4G_coomassie blut.tif]

Figure 4 – source data 1 (panel B)

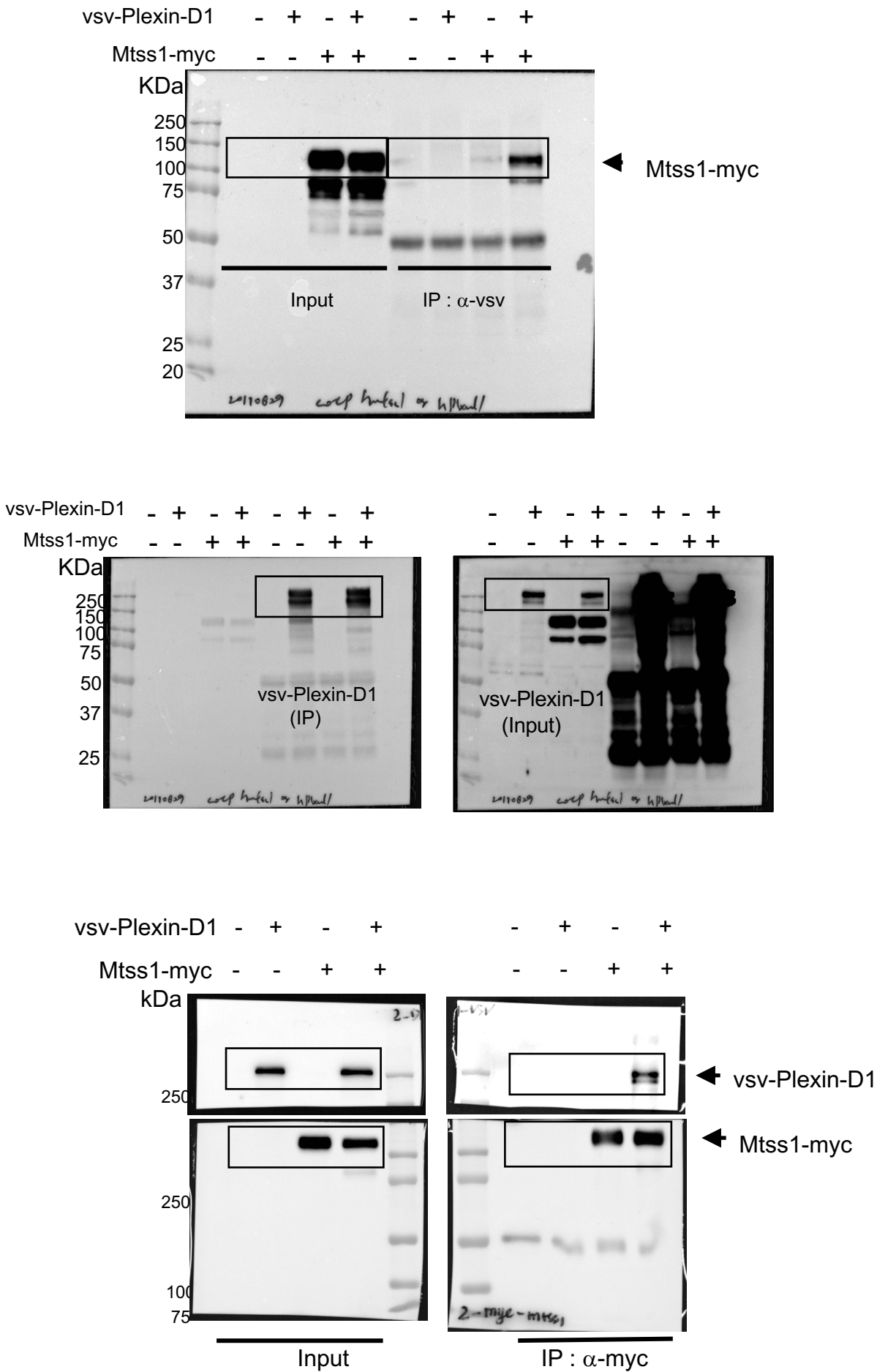

Supplement: Figure 4—source data 1. [file elife-96891-fig4-data1.zip › Figure 4_source data 1/Figure 4_labelled_4B.pdf]

Figure 4 – source data 1 (panel C)

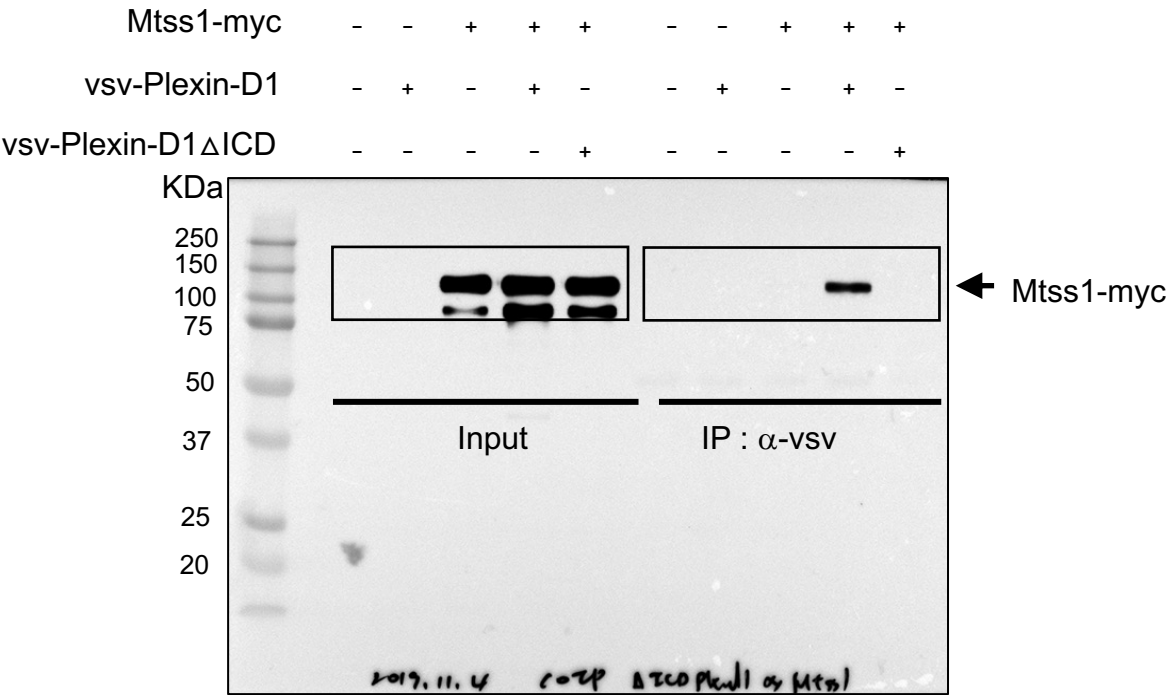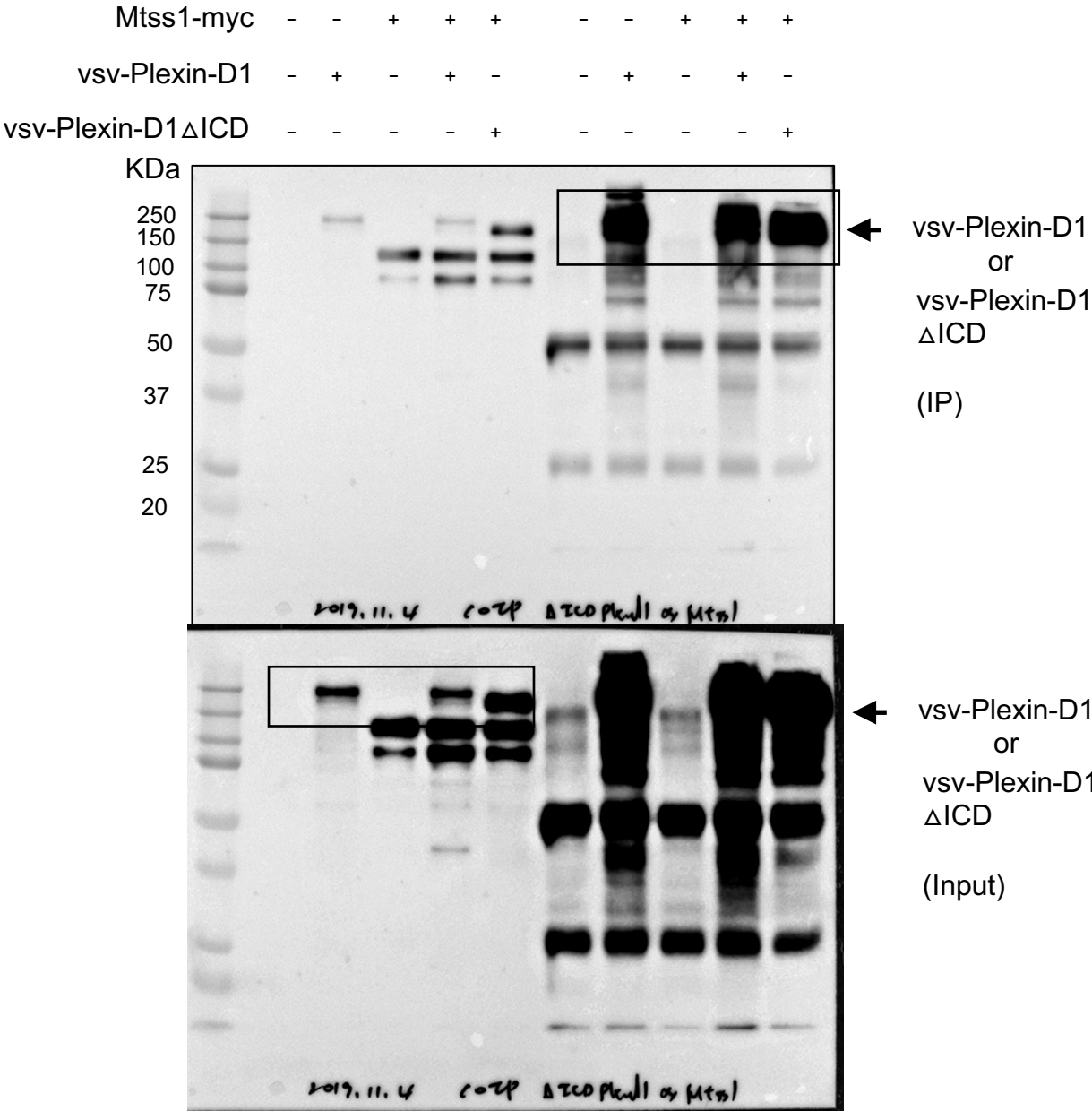

Supplement: Figure 4—source data 1. [file elife-96891-fig4-data1.zip › Figure 4_source data 1/Figure 4_labelled_4C.pdf]

Figure 4 – source data 1 (panel D)

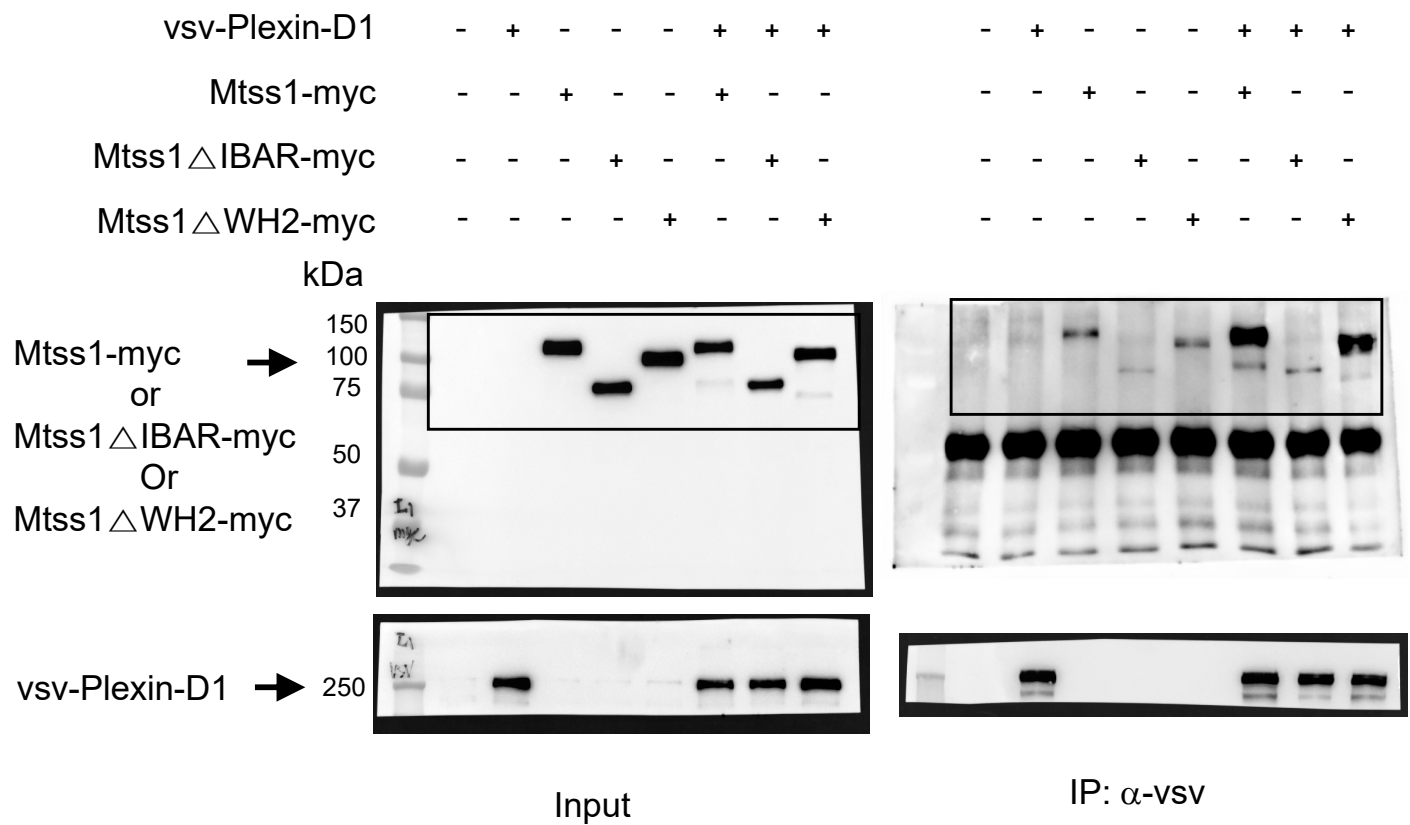

Supplement: Figure 4—source data 1. [file elife-96891-fig4-data1.zip › Figure 4_source data 1/Figure 4_labelled_4D.pdf]

Figure 4 – source data 1 (panel E)

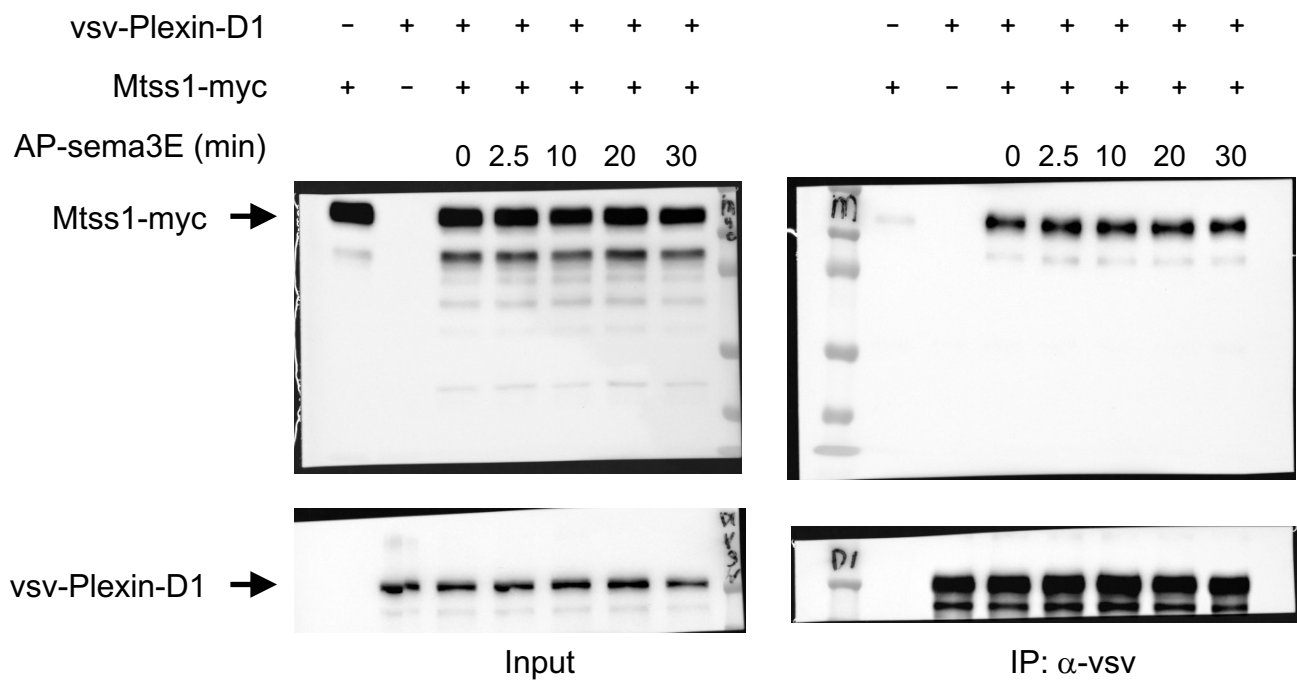

Supplement: Figure 4—source data 1. [file elife-96891-fig4-data1.zip › Figure 4_source data 1/Figure 4_labelled_4E.pdf]

Figure 4 – source data 1 (panel G)

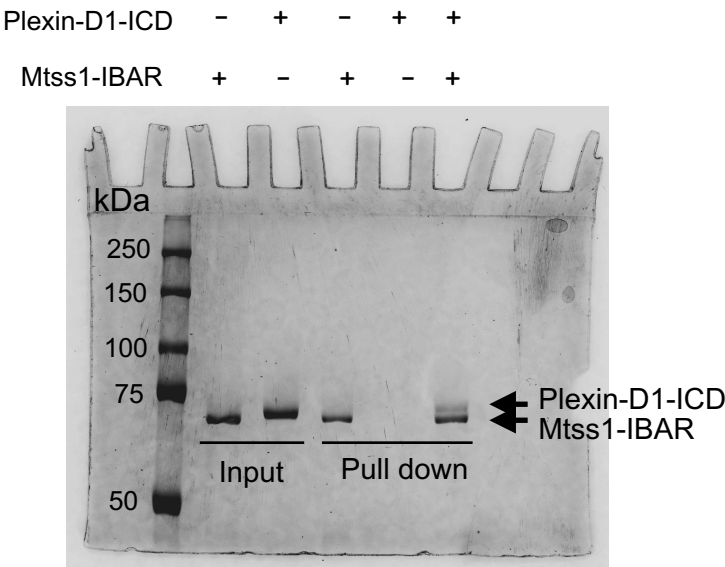

Supplement: Figure 4—source data 1. [file elife-96891-fig4-data1.zip › Figure 4_source data 1/Figure 4_labelled_4G.pdf]

Supplementary Figure 4 – source data 1 (panel A)

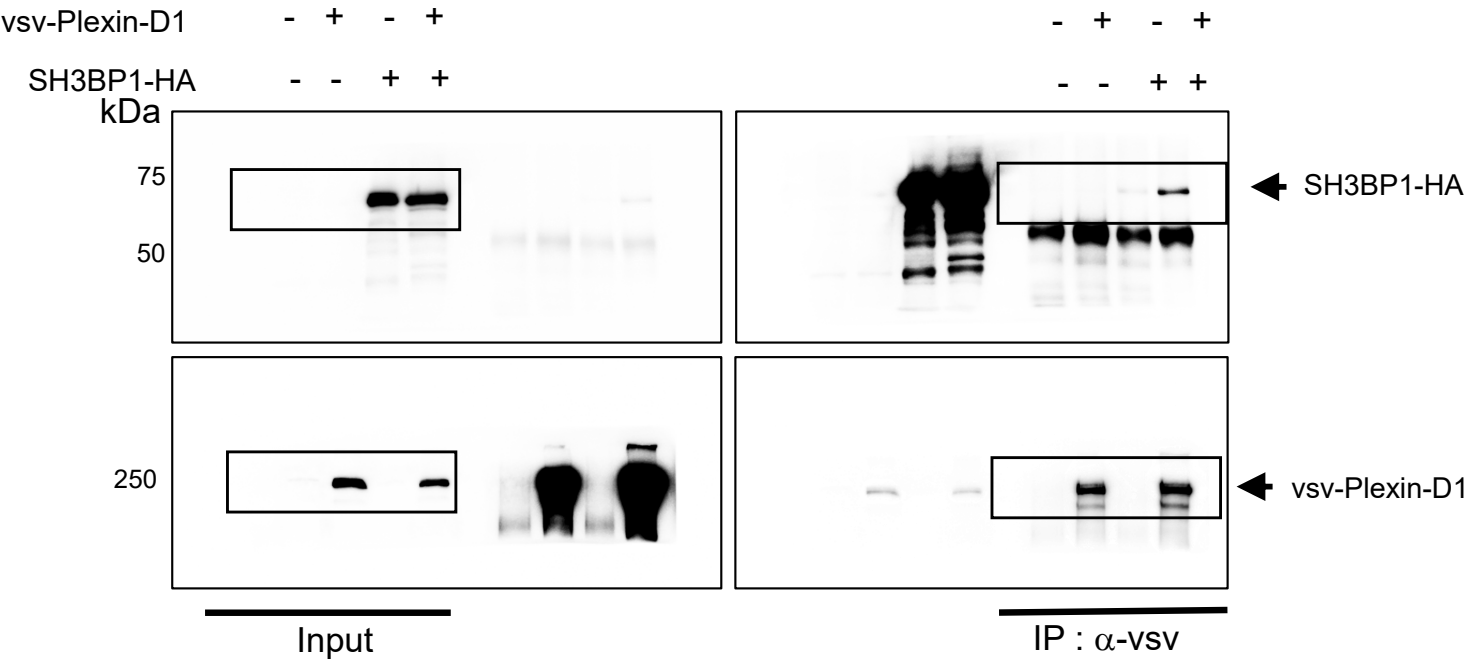

Supplement: Figure 4—figure supplement 1—source data 1. [file elife-96891-fig4-figsupp1-data1.zip › Figure 4_supplementary Figure 4_source data 1/Figure S4_labelled_S4A.pdf]

Supplementary Figure 4 – source data 1 (panel B)

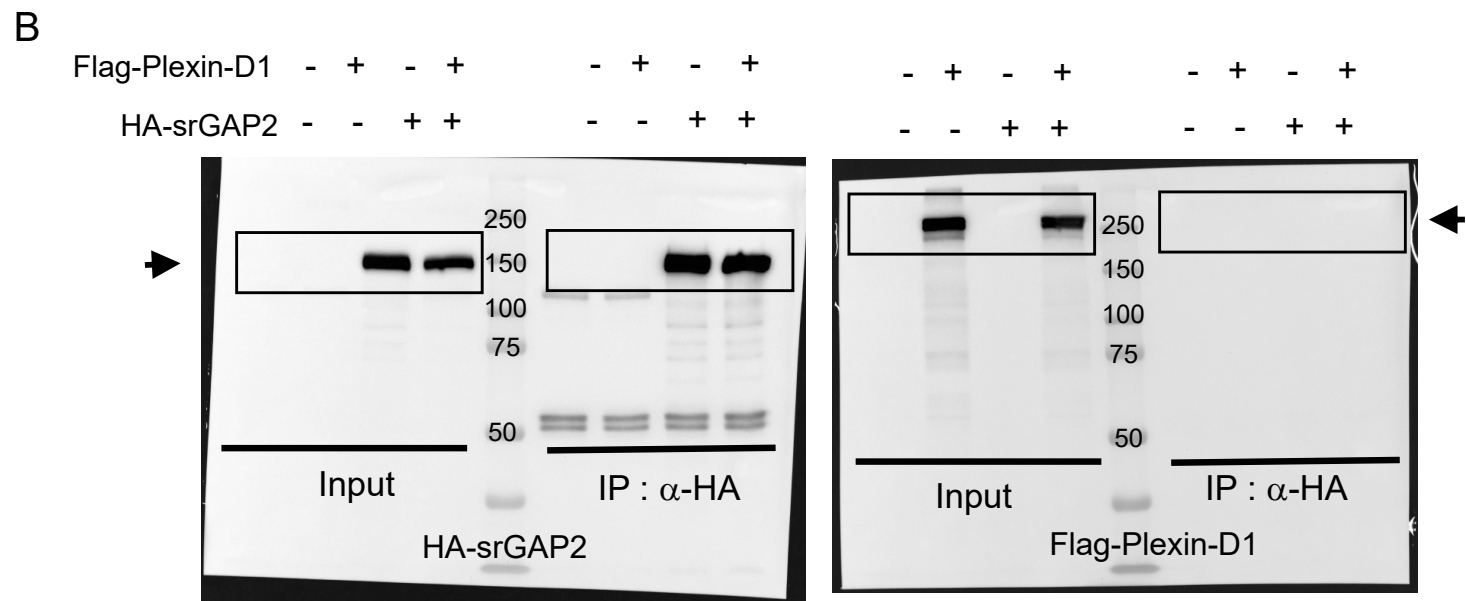

Supplement: Figure 4—figure supplement 1—source data 1. [file elife-96891-fig4-figsupp1-data1.zip › Figure 4_supplementary Figure 4_source data 1/Figure S4_labelled_S4B.pdf]

Supplementary Figure 4 – source data 1 (panel C)

C

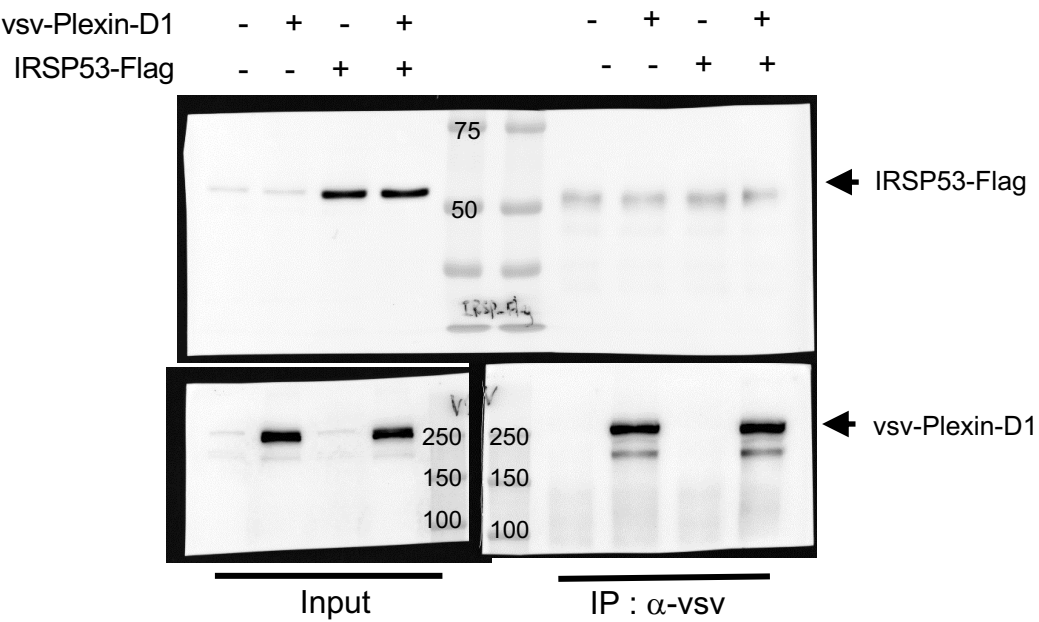

Supplement: Figure 4—figure supplement 1—source data 1. [file elife-96891-fig4-figsupp1-data1.zip › Figure 4_supplementary Figure 4_source data 1/Figure S4_labelled_S4C.pdf]

Supplementary Figure 4 – source data 1 (panel D)

D

|               |   |   |   |   |   |   |   |   |  |   |   |   |   |  |   |   |   |   |
|---------------|---|---|---|---|---|---|---|---|--|---|---|---|---|--|---|---|---|---|
| vsv-Plexin-B2 | - | - | + | + | - | - | + | + |  | - | - | + | + |  | - | - | + | + |
| Mtss1-myc     | - | + | - | + | - | + | - | + |  | - | + | - | + |  | - | + | - | + |

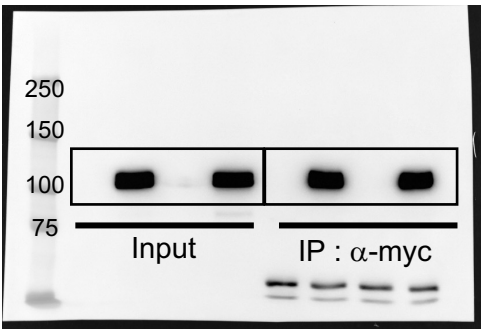

Mtss1-myc

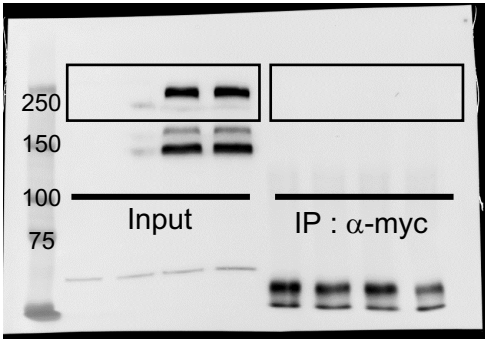

vsv-Plexin-B2

Supplement: Figure 4—figure supplement 1—source data 1. [file elife-96891-fig4-figsupp1-data1.zip › Figure 4_supplementary Figure 4_source data 1/Figure S4_labelled_S4D.pdf]

Supplementary Figure 4 – source data 1 (panel E)

E

|               |   |   |   |   |   |   |   |   |   |   |   |   |
|---------------|---|---|---|---|---|---|---|---|---|---|---|---|
| vsv-Plexin-B3 | - | - | + | + | - | - | + | + | - | - | + | + |
| Mtss1-myc     | - | + | - | + | - | + | - | + | - | + | - | + |

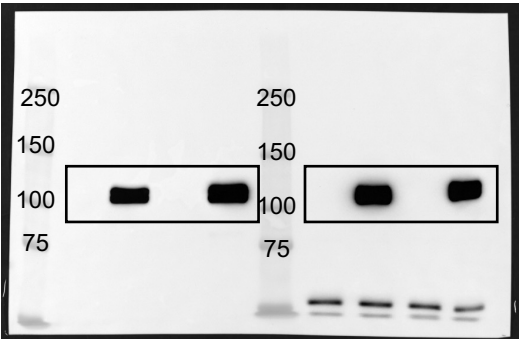

Mtss1-myc

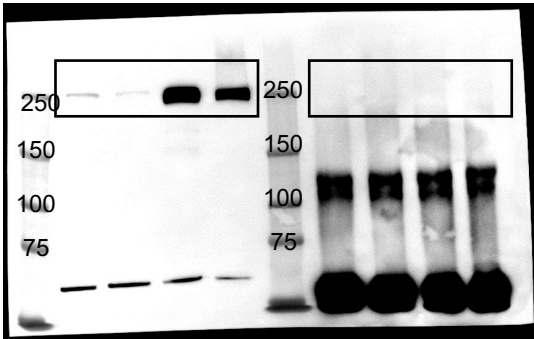

vsv-Plexin-B3

Supplement: Figure 4—figure supplement 1—source data 1. [file elife-96891-fig4-figsupp1-data1.zip › Figure 4_supplementary Figure 4_source data 1/Figure S4_labelled_S4E.pdf]

Supplementary Figure 5 – source data 1 (panel A)

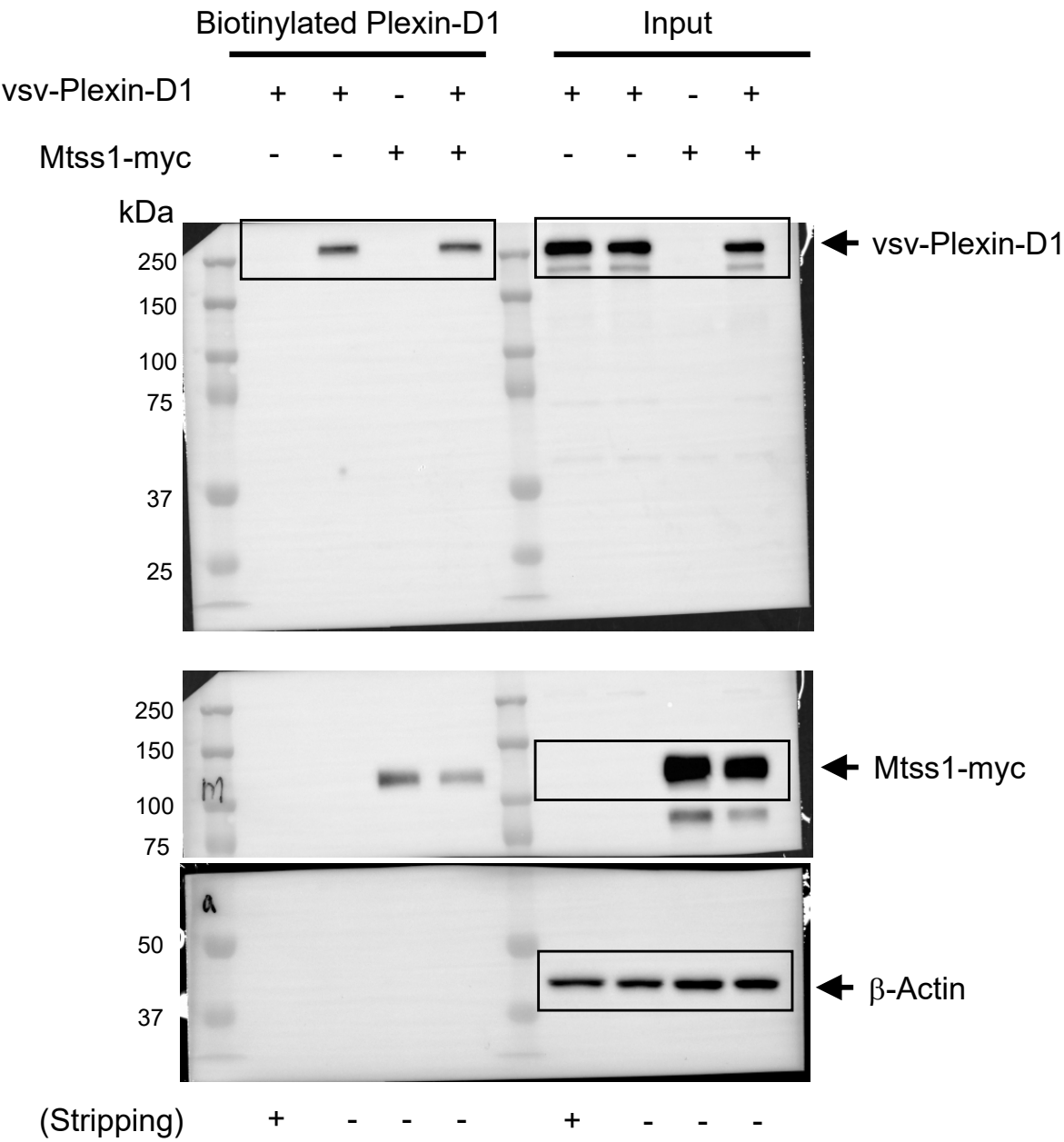

Supplement: Figure 5—figure supplement 1—source data 1. [file elife-96891-fig5-figsupp1-data1.zip › Figure 5_supplementary Figure 5_source data 1/Figure S5_labelled_S5A.pdf]

Supplementary Figure 5 – source data 1 (panel C)

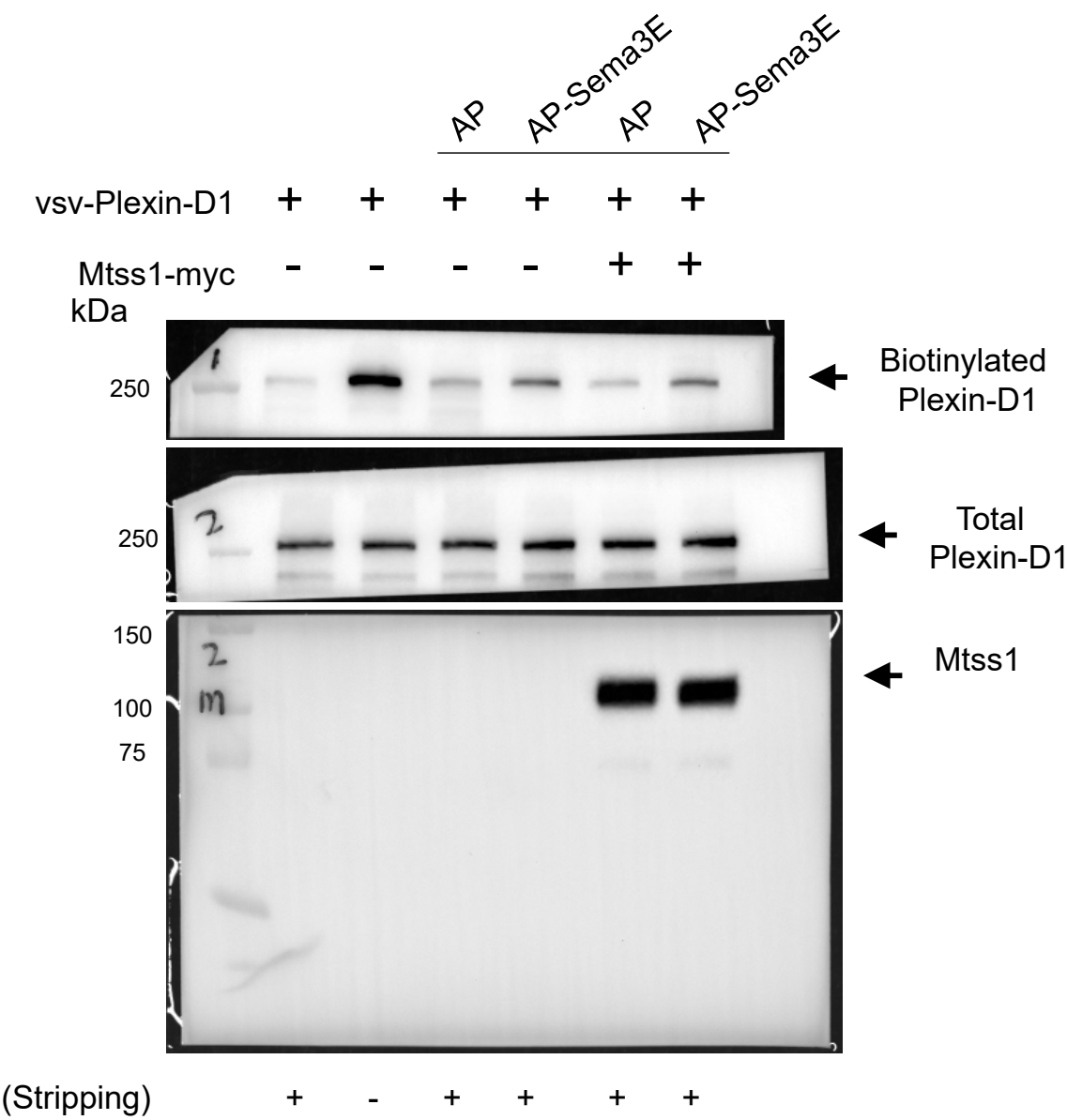

Supplement: Figure 5—figure supplement 1—source data 1. [file elife-96891-fig5-figsupp1-data1.zip › Figure 5_supplementary Figure 5_source data 1/Figure S5_labelled_S5C.pdf]

Supplementary Figure 9 – source data 1 (panel F)

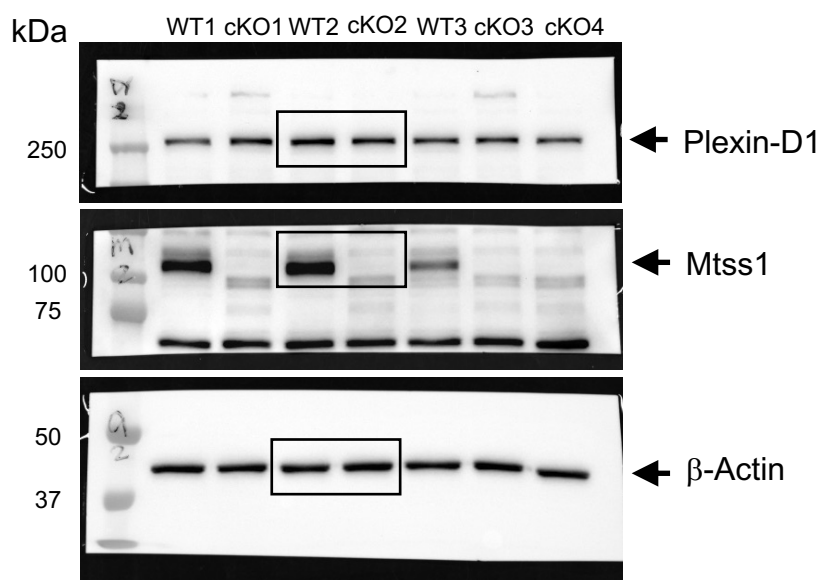

Supplement: Figure 7—figure supplement 3—source data 1. — Western blots shown in Figure 7—figure supplement 3F and H. [file elife-96891-fig7-figsupp3-data1.zip › Figure 7_supplementary Figure 9_source data 1/Figure S9_labelled_S9F.pdf]

Supplementary Figure 9 – source data 1 (panel H)

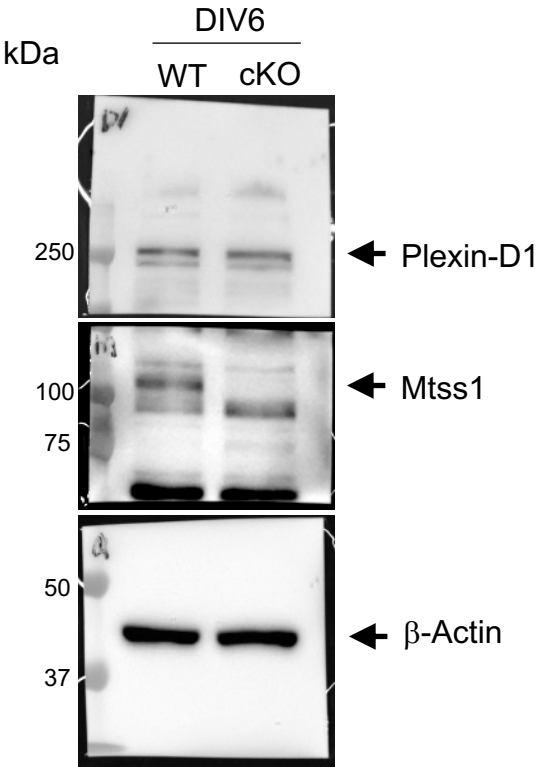

Supplement: Figure 7—figure supplement 3—source data 1. — Western blots shown in Figure 7—figure supplement 3F and H. [file elife-96891-fig7-figsupp3-data1.zip › Figure 7_supplementary Figure 9_source data 1/Figure S9_labelled_S9H.pdf]

Supplementary Figure 11 – source data 1 (panel C)

HUVEC

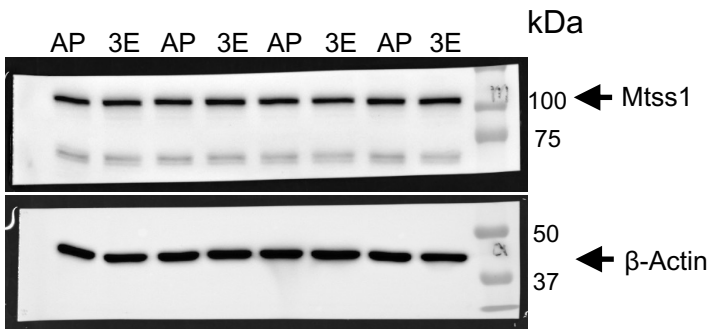

HCMEC

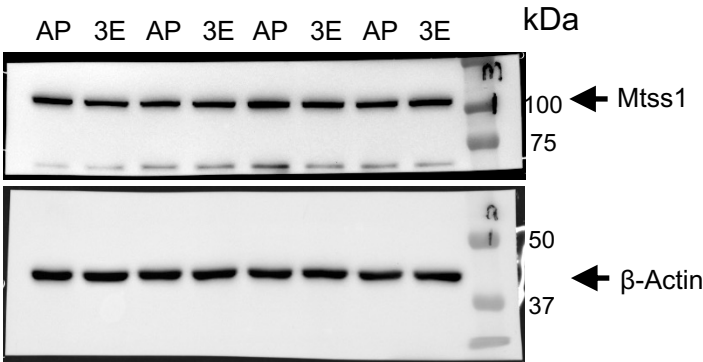

\*

Supplement: Figure 8—figure supplement 1—source data 1. [file elife-96891-fig8-figsupp1-data1.zip › Figure 8_supplementary Figure 11_source data 1/Figure S11_labelled_S11C.pdf]
